# Plant-specific function of H3K9me3 as a permissive chromatin mark during Arabidopsis seed germination

**DOI:** 10.3389/fpls.2026.1785818

**Published:** 2026-03-12

**Authors:** Jae-Wook Yoon, Min-Jeong Kang, Hongshi Jin, Yoo-Sun Noh, Bosl Noh

**Affiliations:** 1 School of Biological Sciences, Seoul National University, Seoul, Republic of Korea; 2 Research Center for Plant Plasticity, Seoul National University, Seoul, Republic of Korea

**Keywords:** Arabidopsis, H3K9me3, histone methylation, seed germination, transcriptional reprogramming

## Abstract

Seed dormancy and germination are sequential phases, both tightly regulated by environmental and hormonal cues to ensure germination under favorable conditions. While hormonal and transcriptional regulators are well characterized, the epigenetic mechanisms governing these transitions are less understood. We profiled H3K4me3, H3K27me3, and H3K9me3 in the seeds of *Arabidopsis thaliana* Columbia-0 and Cape Verde Islands, representing shallow and deep dormancy, respectively, at freshly harvested (FH), after-ripened (AR), and germination-stimulated (GS) states. H3K4me3 and H3K9me3 co-localized in euchromatic, transcriptionally active regions, whereas H3K27me3 occupied repressive domains. Histone methylation landscapes were stable from FH to AR but showed a marked H3K9me3 increase from AR to GS in association with the activation of genes related to translation, energy metabolism, and cell division. Co-marking by H3K4me3 and H3K9me3 correlated with highest transcript levels. During dormancy release, AR-repressed genes, including *DOG1*, showed targeted reductions in H3K4me3 and H3K9me3 levels. These findings support a plant-specific role of H3K9me3 as an active or permissive chromatin mark promoting transcriptional reprogramming during germination.

## Introduction

Seed germination is a critical developmental transition in the plant life cycle, as its proper timing is essential for ensuring survival and sustained growth in sessile organisms. Accordingly, germination is tightly regulated by a complex interplay of internal and environmental cues to occur only under favorable conditions. In some species, such as *Arabidopsis thaliana*, seeds may fail to germinate even when environmental conditions are favorable, due to the presence of seed dormancy (Bewley, 1997; Hilhorst, 2008; Li and Foley, 1997). This primary seed dormancy is progressively developed during seed maturation and becomes fully established in association with desiccation tolerance at the end of maturation. Dormancy is alleviated over time during dry storage of mature seeds, a process known as after-ripening, and can be further reduced by cold and imbibition treatment, referred to as stratification. Germination is initiated only after dormancy is sufficiently released, likely reflecting the establishment of a cellular environment permissive for transcriptional and metabolic reactivation (Weitbrecht et al., 2011).

Understanding the molecular mechanisms that govern the transition from dormancy to germination is of fundamental importance in both seed biology and agriculture. Numerous studies have sought to elucidate the regulatory pathways underlying this process. Abscisic acid (ABA) promotes dormancy during seed maturation and inhibits germination of dormant seeds (Kanno et al., 2010; Kinoshita et al., 2010; Kucera et al., 2005), whereas gibberellins (GAs) antagonize ABA action to promote germination by inducing key gene-regulatory networks required for germination (Iwasaki et al., 2022; Lee et al., 2002; Liu and Hou, 2018; Piskurewicz et al., 2008). *DELAY OF GERMINATION1* (*DOG1*) is a key regulator of seed dormancy, and natural variations in *DOG1* have reported to contribute to differences in dormancy depth among Arabidopsis accessions (Finch-Savage and Footitt, 2017; Footitt et al., 2020). While it is now revealed that hormonal balance and intricate molecular and biochemical networks are involved in dormancy and germination, the precise molecular basis of these processes is yet to be fully characterized.

Histone methylation is a key epigenetic modification that influences transcription by providing binding surfaces for reader proteins, which in turn recruit transcription factors, chromatin modifiers, and RNA polymerase machinery (Haider and Farrona, 2024). Depending on the specific lysine or arginine residue and methylation state, histone methylation can either activate or repress transcription. In plants, trimethylation of histone H3 at lysine 4 (H3K4me3) and lysine 36 (H3K36me3) are generally associated with gene activation (Li et al., 2015; Zhang et al., 2009), whereas H3K27me3 and plant-specific H3K9me2 are linked to transcriptional repression (Deleris et al., 2012; Lippman et al., 2004; Roudier et al., 2011). The function of H3K9me3 in plants, however, remains less well understood. Unlike in animals, where it is a hallmark of constitutive heterochromatin and transcriptional silencing (Bannister et al., 2001; Bulut-Karslioglu et al., 2014; Peters et al., 2001), H3K9me3 in plants is often enriched in euchromatic regions and detected over transcriptionally active genes, with limited enrichment at transposable elements (Naumann et al., 2005; Roudier et al., 2009; Veiseth et al., 2011).

Several studies have implicated histone methylation in regulating seed dormancy and germination. For example, the Arabidopsis H3K4 demethylase LSD1-like1 (LDL1) and LDL2 reduce seed dormancy by downregulating *DOG1* and ABA signaling-related genes such as *ABI3* (Zhao et al., 2015). The H3K27me3 demethylase RELATIVE OF EARLY FLOWERING6 (REF6) promotes germination by facilitating the activation of ABA catabolism genes such as *CYTOCHROME P450, FAMILY707, SUBFAMILY A, POLYPEPTIDE1* (*CYP707A1*) and *CYP707A3*, and by relieving repression of endosperm-expressed genes that may contribute to dormancy (Chen H. et al., 2020). The B3-domain transcriptional repressors VIVIPAROUS1/ABI3-LIKE1 (VAL1) and VAL2, which interact with the H3K27me3 methyltransferase CURLY LEAF (CLF) and the reader LIKE HETEROCHROMATIN PROTEIN1 (LHP1), suppress seed dormancy and promote seedling establishment by repressing *DOG1* expression (Chen N. et al., 2020). Although these studies underscore the importance of histone methylation at specific dormancy-related loci, it remains unclear whether these marks are dynamically regulated during seed-state transitions, or whether genome-wide changes in histone methylation contribute to the broader transcriptional reprogramming associated with dormancy release and germination.

Advances in genomic and epigenomic research tools such as RNA sequencing (RNA-seq) and chromatin immunoprecipitation sequencing (ChIP-seq) have enabled systematic investigation of gene expression and chromatin modifications across plant developmental transitions (Le et al., 2025). However, genome-wide studies of histone methylation dynamics during dormancy release and germination remain limited in seed biology. Beyond histone methylation, the roles of other histone modifications and epigenetic mechanisms—and their coordination with transcriptomic reprogramming—remain largely unexplored in this context. Recent reports have begun to address this gap, examining chromatin accessibility, *de novo* RNA synthesis, H3.3 histone variant deposition, and H3K27me3 dynamics during seed germination (Sato et al., 2021; Tremblay et al., 2024; Wang et al., 2023; Zhao et al., 2022). Yet to date, comprehensive genome-wide studies of the after-ripening phase are still lacking, with only one study so far integrating DNA methylation, small RNA, and transcriptomic profiles in dormant and after-ripened seeds (Lee et al., 2023).

In this study, we focused on and examined the dynamics of three histone methylation marks—H3K4me3 (a representative active mark), H3K27me3 (a representative repressive mark), and H3K9me3 (a mark whose function is unclear in plants) (Veiseth et al., 2011; Xu and Jiang, 2020)—across three distinct seed states: freshly harvested (FH), after-ripened (AR), and germination-stimulated (GS). We integrated these epigenomic profiles with transcriptome data to assess whether transcriptional reprogramming during seed-state transitions is coordinated with or regulated by epigenomic reprogramming. To investigate seed-state-dependent histone methylation and transcriptomic dynamics in contrasting dormancy backgrounds and examine whether these patterns are shared or accession-specific, we performed parallel analyses in two *Arabidopsis thaliana* accessions: Columbia-0 (Col), which exhibits shallow dormancy, and Cape Verde Islands (Cvi), which demonstrates deep dormancy. Through these analyses, we found that while histone methylation landscapes remained largely stable during the FH-to-AR transition, a substantial increase in H3K9me3 occurred during the AR-to-GS transition. H3K9me3 was predominantly localized to transcriptionally active euchromatic regions and showed a positive correlation with gene expression across seed states. These findings indicate that H3K9me3 is likely to act as a key epigenetic regulator of transcriptional reprogramming during the AR-to-GS transition, potentially promoting the expression of genes required for germination. Our study also provides strong evidence that H3K9me3 has a plant-specific role as an active or permissive epigenetic mark associated with transcription.

## Materials and methods

### Plant materials and growth conditions

*Arabidopsis thaliana* accessions Columbia-0 (Col) and Cape Verde Islands (Cvi) were used in this study. Plants were grown under standard long-day conditions (16h light/8h dark) at 22 °C. Freshly harvest (FH) seeds were obtained from yellow-green siliques 14–16 days after pollination (DAP). After-ripened (AR) seeds were collected from yellow-brown dehiscent siliques, followed by the subsequent storage for two weeks (w) for Col and 60 days (d) for Cvi at room temperature. Germination-stimulated (GS) seeds were prepared by plating AR seeds on 1/2 Murashige and Skoog (MS) medium, followed by a 3-d imbibition at 4°C, a 2-hour (hr) white-light exposure, and a 24-hr dark incubation at 22 °C.

### Chromatin immunoprecipitation followed by sequencing and analysis

Seed chromatin was isolated for native-ChIP as previously described by Müller et al (Muller et al., 2012). with the following modifications. Whole seeds rather than isolated embryos were used for chromatin extraction. Chromatin was fragmented using MNase (NEB, MS247S) by incubating at 37 °C for 20 minutes (min), extending the incubation time from the 8 min described in the original protocol. Mnase digestion generated predominantly mono- to tri-nucleosome-sized fragments (~150–500 bp), and fragment size distribution was monitored by agarose gel electrophoresis. For immunoprecipitation of histone protein-DNA complexes, the following antibodies were used: α-H3K4me3 (Millipore, 07-473), α-H3K9me3 (Millipore, 07-442), and α-H3K27me3 (Millipore, 07-449). ChIPed DNAs were purified by the MinElute PCR Purification Kit (Qiagen, 28006), followed by Proteinase K (Roche, 03115828001) treatment.

ChIP-seq libraries were constructed using the TruSeq Library Preparation Kit (Illumina, IP-202-1012) and sequenced on the Illumina HiSeq4000 platform by Macrogen (Seoul, South Korea). Raw reads were trimmed and quality-checked using Trimmomatic (v0.39) (Bolger et al., 2014) with the following parameters: “PE -phred33 ILLUMINACLIP: TruSeq3-PE.fa:2:30:10 LEADING:3 TRAILING:3 SLIDINGWINDOW:4:15 MINLEN:36”. Trimmed reads were mapped to the nuclear genome of Arabidopsis using Bowtie2 (v2.5.1) (Langmead and Salzberg, 2012) with the options “-p 40 -I 0 -X 500”. For Col, the TAIR10 reference genome (Reiser et al., 2024) was used. For Cvi, a pseudo-Cvi genome was generated by substituting Col SNPs in the TAIR10 with publicly available Cvi SNPs (Jiao and Schneeberger, 2020). Duplicate reads were removed using Picard MarkDuplicates (v2.18.29) (McKenna et al., 2010) with the options “--REMOVE_DUPLICATES=true ASSUME_SORTED=true QUIET=true VALIDATION_STRINGENCY=LENIENT”. Only uniquely mapped reads were used for further analysis.

Histone methylation peaks were identified using MACS2 callpeak (v2.2.7.1) (Zhang et al., 2008) with the parameters “-f BAMPE --max-gap 10 -B -q 0.01 -g 118078569 (116882681 for Cvi samples using the pseudo-Cvi Tair10)”. Resulting peaks were filtered against the Arabidopsis greenscreen regions (Klasfeld et al., 2022) to remove artifactual signals using BEDTools (v2.31.1) (Quinlan and Hall, 2010) intersect with the “-v” option. For comparative analysis, combined peak files across FH, AR, and GS states for each histone methylation mark (H3K4me3, H3K9me3, or H3K27me3) were generated using BEDTools merge.

H3K4me3 and H3K9me3 peaks were annotated against a modified *Arabidopsis thaliana* gene annotation file in which gene bodies were defined as 50% of region downstream from the transcription start sites (TSSs). Annotation was performed using BEDTools intersect with the option “-f 0.3”. Peaks not overlapping with this modified annotation file were further annotated using the option “-F 0.3”, and the two resulting sets were subsequently merged. H3K27me3 peaks were annotated against the standard *Arabidopsis thaliana* gene annotation file.

BigWig files were generated using deepTools bamCompare (v3.5.5) (Ramirez et al., 2016) with the parameters “--scaleFactorsMethod None --operation subtract -bs 10 --normalizeUsing CPM -bl”, to produce input-subtracted signal tracks while excluding the Arabidopsis greenscreen regions (Klasfeld et al., 2022) to reduce background noise. These bigWig files were subsequently used to calculate signal coverage across defined genomic regions using computeMatrix, and to generate heatmaps and metaplots using plotHeatmap and plotProfile, respectively. Visualization of bigWig files was performed using the IGB browser (v10.1.0) (Freese et al., 2016).

Histone methylation enrichment scores were calculated from the bigWig files using deepTools multiBigwigSummary (v3.5.5) (Ramirez et al., 2016) to obtain signal values in given BED-format regions or across genomic 10 kb-sized bins. The resulting matrices were used as input for plotCorrelation (Galaxy Version 3.5.4+galaxy0) (Ramirez et al., 2016) with Spearman’s rank correlation to generate heatmaps representing pairwise correlation scores for each histone methylation mark across seed states or between different histone marks within each seed state.

For the identification of differential peaks, the bigWig files were converted to BedGraph format using UCSC bigWigToBedGraph (v469) (Perez et al., 2025) with default settings, followed by differential peak calling using MACS2 bdgdiff (v2.2.7.1) (Zhang et al., 2008). Identified differential peaks were annotated using the same approach as used for the peak annotation.

### RNA sequencing analysis

RNA-seq raw reads (SRA accession number: PRJNA835511) (Lee et al., 2023) were downloaded from NCBI and processed as described for ChIP-seq except for the alignment step. Alignment of trimmed reads to the appropriate reference genomes was performed using HISAT2 (v2.2.1) (Kim et al., 2019) with the options “-p 40 --dta –fr”. Transcript assembly and quantification were conducted using StringTie (v2.2.3) (Pertea et al., 2015) on aligned BAM files using the Araport11 genome annotation (Oct. 2023 version) as a reference, with the options “-G -e”. Gene- and transcript-level count matrices were subsequently generated using the *prepDE.py* script. Differential gene expression analysis was performed using limma-voom (Galaxy Version 3.50.1+galaxy0) (Law et al., 2014; Smyth, 2005), edgeR (Galaxy Version 3.36.0+galaxy4) (Liu et al., 2015; Robinson et al., 2010), and DEseq2 (Galaxy Version 2.11.40.8+galaxy0) (Love et al., 2014), applying an expression filter defined as CPM ≥ 1 in more than two samples. Differentially expressed genes (DEGs) with log_2_(fold change) ≥ 1 and FDR < 0.05 across all methods were considered as DEGs.

For visualization, bigWig files were generated using deepTools bamCoverage (v3.5.5) (Ramirez et al., 2016) with the parameters “--scaleFactorsMethod None -bs 10 --normalizeUsing RPKM”. For quantification of gene expression levels, the log_2_-transformed normalized CPM values from edgeR (TMM-normalized outputs) were reverse-transformed and divided by gene lengths to produce normalized RPKM (nRPKM) values.

Differential expression analysis of capped-small RNA sequencing (csRNA-seq) data (Tremblay et al., 2024) was performed using raw count matrices provided by the authors (SRA accession number: GSE250329) (Tremblay et al., 2024). EdgeR was used to identify differentially expressed transcription sites (DE TSSs) based on a threshold of log_2_(fold change) ≥ 1 and FDR < 0.05. DE TSSs were annotated by the TSS information provided in the **Supplementary Data 1** of Tremblay et al., 2024, and the resulting gene list was used to identify genes overlapping with the C1 to C5 cluster genes for **Supplementary Figure 10**.

### Clustering of differentially expressed genes

Clustering of genes differentially expressed during seed state transitions was performed using normalized RPKM values (described above), applying *k*-means clustering (using one minus Pearson correlation distance) in Morpheus, as mentioned in figure legends.

### Gene ontology analysis and functional clustering

GO analysis of *k*-means clustered genes was conducted using the ShinyGO (v0.8.0) (Ge et al., 2020). Comparative GO analysis across gene sets was performed using the compareCluster function implemented in the ClusterProfiler package (v4.12.6) (Wu et al., 2021; Yu et al., 2012). Functional annotation clustering of DEGs during the FH-to-AR transition was conducted using DAVID (Huang da et al., 2009; Sherman et al., 2022).

### Deep RNA-seq

Two biological replicates of deep RNA-seq libraries were prepared following the manufacturer’s instructions (Illumina) by using total RNAs isolated from Cvi FH and AR seeds. About 60 ng/μl per sample was obtained after NGS library construction for paired-end strand-specific deep-sequencing. The libraries were validated using Agilent 2100 Bioanalyzer. Transcriptome sequencing was performed on the Illumina TruSeq Stranded HiSeq 2000 platform by Macrogen (Seoul, South Korea). More than 20 Gbp reads per sample condition were obtained. After trimming artifacts and processing with Trimmomatic, clean data with 92% or higher for Phred quality score were obtained.

### RNA extraction and reverse transcription followed by real-time quantitative PCR analysis

Total RNA was extracted as previously described (Meng and Feldman, 2010), treated with DNase (Qiagen, 79254), and purified using the RNeasy Mini Kit (Qiagen, 74106). 3 µg of total RNA per sample was reverse-transcribed using oligo(dT) primers and M-MuLV Reverse Transcriptase (Thermo Scientific, EP0352). qPCR was performed in the Rotor-Gene Q real-time PCR system (QIAGEN) using TOPreal SYBR Green qPCR PreMIX (Enzynomics, RT500). The resulting synthesized cDNA was analyzed using the standard curve method. The amplification values were normalized to the expression of the reference gene *PP2A*. RT–qPCR analyses were conducted using three independent biological replicates, each analyzed with three technical replicates. The mean of the technical replicates was used for quantification unless clear outliers were identified and excluded. Results were presented as mean ± standard error (SE) from three biological replicates. The sequences of primers used for RT-qPCR are listed in **Supplementary Table 1**.

### ChIP assay

Chromatin extraction and Mnase-based fragmentation were performed as described for ChIP-seq. qPCR was performed on ChIPed DNAs using primers listed in **Supplementary Table 1**. Three independent biological replicates were analyzed, each with three technical qPCR replicates. Enrichment was quantified using the comparative Ct method (2^-ΔΔCt^) (Livak and Schmittgen, 2001), with normalization to input DNA. ChIP–qPCR assays were conducted using independently prepared samples separate from those used for ChIP-seq.

## Results and discussion

### Genome-wide profiling of H3K4me3, H3K9me3, and H3K27me3 in dormant, after-ripened, and germination-stimulated seeds

**Figure 1:**
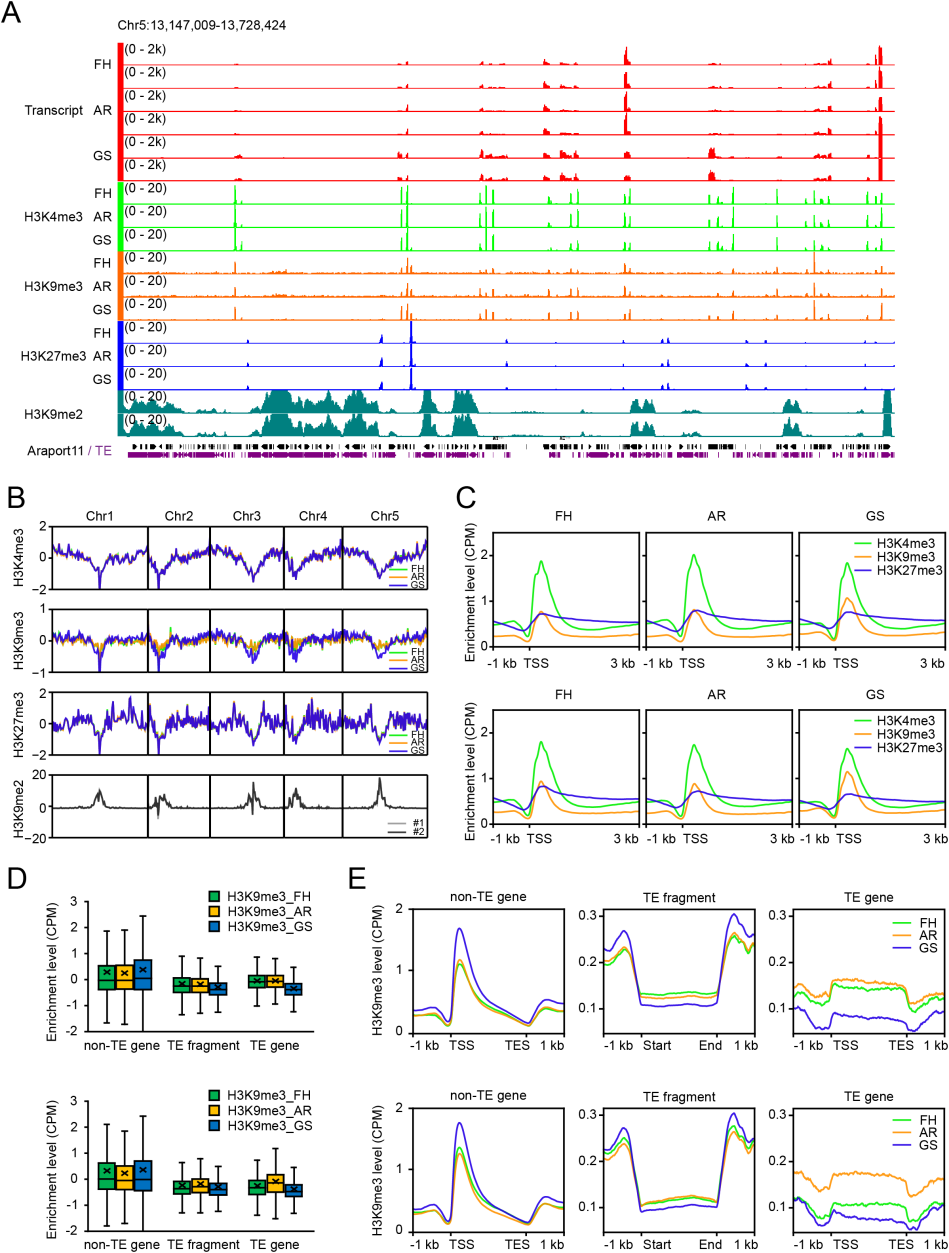
Genome-wide distributions of H3K4me3, H3K9me3 and H3K27me3 in Arabidopsis seed. **(A)** Genome browser view of RNA-seq and histone methylation ChIP-seq signal tracks in seeds across a representative Col genomic region. For the RNA-seq track, published seed RNA-seq data (SRA accession: PRJNA835511; Lee et al., 2023) were used. For comparison, signal tracks of previously published H3K9me2 ChIP-seq data from Col seedlings (SRA accession: PRJNA762490; Zhao L et al., 2022) are also shown. Araport11 gene models are displayed as black bars and lines, and transposable elements (TEs) are displayed as purple bars. **(B)** Distribution of H3K4me3, H3K9me3, and H3K27me3 in seeds along chromosomes 1–5 of Col. H3K9me2 signal is also displayed as a pericentromeric marker. **(C)** Metaplots of H3K4me3, H3K9me3, and H3K27me3 across regions spanning 1 kb upstream to 3 kb downstream of transcription start sites (TSS) of all genes in Col (upper) and Cvi (lower). **(D, E)** Box plots **(D)** showing H3K9me3 levels and metaplots **(E)** showing the distribution pattern of H3K9me3 enrichment over non-TE genes, TE fragments, and TE genes in Col (upper) and Cvi (lower). Histone methylation levels for **(B–E)** are presented as input-subtracted CPM values (see Materials and Methods). In **(D)**, horizontal line and cross within each box indicate the median and mean, respectively; boxes span the interquartile range (25th–75th percentile), and whiskers extend to adjacent values (i.e., the most extreme data points within 1.5 x the interquartile range).

To investigate whether the epigenetic landscapes of the genome in seeds are associated with the germination potential, we examined the genome-wide enrichments of H3K4me3, H3K9me3, and H3K27me3 in Arabidopsis seeds at dormant freshly-harvested (FH), dormancy released after-ripened (AR), and germination-stimulated (GS) states of the two *Arabidopsis thaliana* accessions, Col and Cvi, each representing shallow and deep dormancy, respectively. ChIP-seq analyses revealed different patterns of distributions for the three histone methylation marks. H3K4me3 was enriched mainly at genic regions within euchromatin, consistent with its well-known role as a transcription active mark, while H3K27me3 displayed broad peaks over genic and intergenic regions, reflecting its role as transcription repression mark (**Figures 1A–C**). These distribution patterns were similar to those reported in other tissues (Foroozani et al., 2021; Li et al., 2015; Roudier et al., 2011; Zhang et al., 2009, 2007) and across diverse organisms (Bannister and Kouzarides, 2011; Margueron and Reinberg, 2011; Zhou et al., 2011).

H3K9me3 exhibited a markedly different pattern from the ones in animals. In animals, H3K9me3 is a canonical mark of constitutive heterochromatin and is predominantly enriched at pericentromeric and telomeric regions as well as within large repetitive elements (Bulut-Karslioglu et al., 2012; Nicetto and Zaret, 2019; Udugama et al., 2015). By contrast, consistent with previous studies in plants (Charron et al., 2009; Naumann et al., 2005), our study showed that H3K9me3 was predominantly enriched at euchromatic regions and often colocalized with H3K4me3 and transcribed genes (**Figure 1A**). Whole-genome plots further supported these findings, showing that H3K4me3 and H3K9me3 were enriched in euchromatin, whereas the heterochromatin-associated mark H3K9me2 was confined to pericentromeric heterochromatin (**Figure 1B**). Histone methylation metaplot analyses and the genomic feature-based quantification revealed that H3K4me3 and H3K9me3 were markedly enriched near transcription start sites (TSSs), whereas H3K27me3 exhibited broad deposition across gene bodies (**Figure 1C**; **Supplementary Figure 1**, **S2**). These TSS-centered H3K4me3 and the broad deposition of H3K27me3 across gene bodies are consistent with their established roles in transcription-permissive and Polycomb-repressive contexts, respectively. Within this genomic framework, the TSS-centered enrichment of H3K9me3 suggests that, in Arabidopsis seeds, H3K9me3 may function in a transcription-permissive chromatin context rather than as a canonical heterochromatic mark observed in animals.

To further investigate the genomic context of H3K9me3 deposition, we analyzed its distribution across non-TE genes, TE genes, and TE fragments. Our data showed that H3K9me3 was enriched predominantly in non-TE genes, with significantly lower levels in TE genes and TE fragments (**Figures 1D, E**). This finding indicates that in Arabidopsis, H3K9me3 is primarily associated with actively transcribed coding genes, rather than with silenced repetitive elements. Notably, the profiles of H3K9me3 peaks in TE genes and TE fragments differed from those in non-TE genes, resembling the broad deposition patterns of H3K27me3, suggesting a functional specificity of H3K9me3 in actively transcribed regions of the genome. Taken together, these results revealed the distinct genomic distributions of different histone methylation marks during seed-state transitions and support the emerging view of the role of H3K9me3 in euchromatin in plants.

### Combinatorial occurrence of histone methylation marks

**Figure 2:**
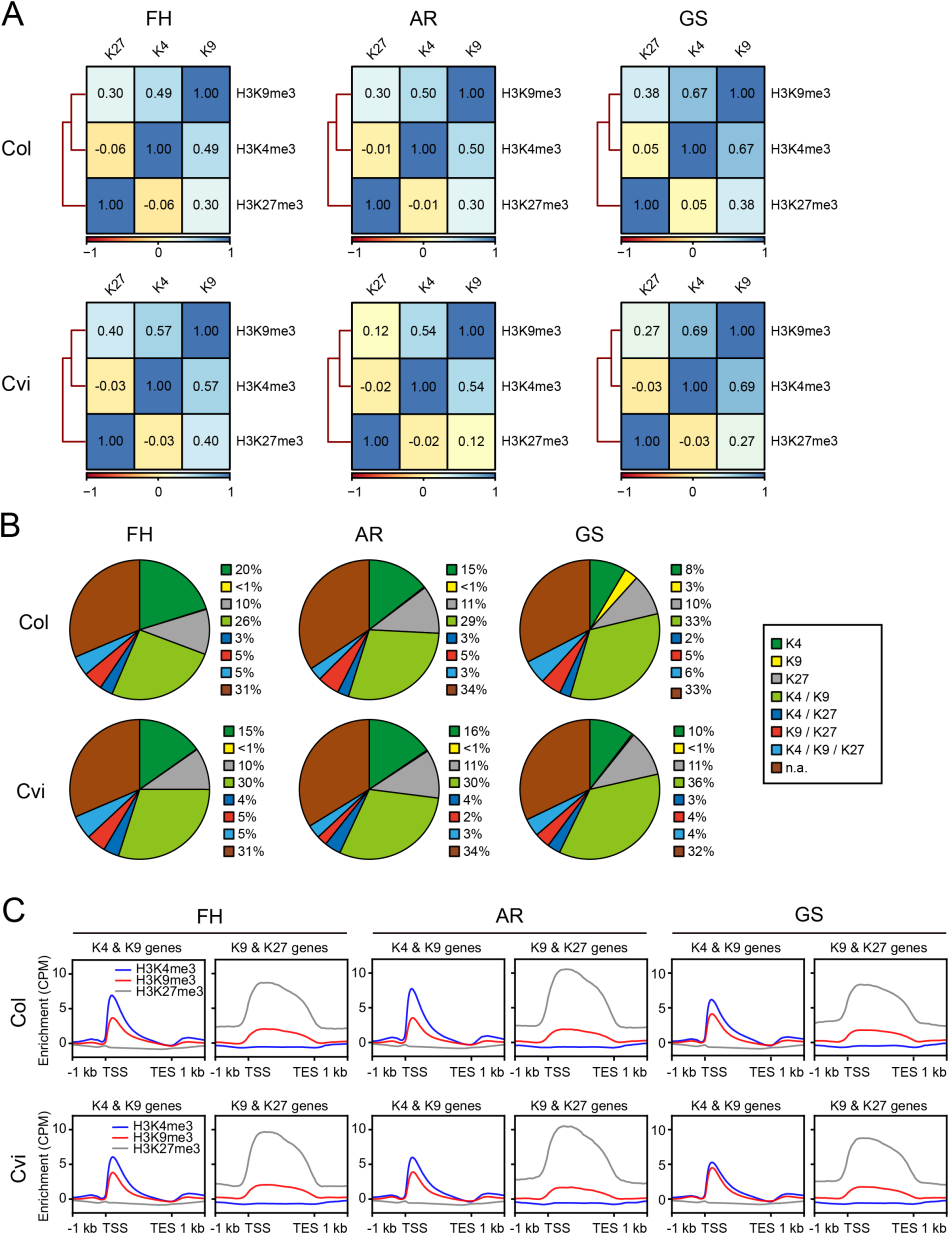
Correlation between histone methylation marks. **(A)** Heatmaps showing hierarchical clustering based on Spearman’s rank correlation of H3K4me3, H3K9me3, and H3K27me3 in Col and Cvi seeds. Correlation coefficients were calculated using CPM values (see Materials and Methods) in 10 kb-bins across the whole genomes. K27, H3K27me3; K4, H3K4me3; K9, H3K9me3. **(B)** Pie charts showing the relative proportions of genes carrying individual or combinatorial histone marks. K4, K9, and K27 indicate genes marked solely with H3K4me3, H3K9me3, or H3K27me3, respectively; K4/K9, K4/K27, and K9/K27 indicate genes marked with H3K4me3 and H3K9me3, H3K4me3 and H3K27me3, or H3K9me3 and H3K27me3, respectively; K4/K9/K27 indicates genes marked with H3K4me3, H3K9me3, and H3K27me3. n.a. denotes genes lacking all three histone marks. **(C)** Metaplots showing the enrichments of H3K4me3, H3K9me3, and H3K27me3 over genes marked with H3K4me3 and H3K9me3 but not with H3K27me3, or over genes marked with H3K9me3 and H3K27me3 but not with H3K4me3, in Col (upper) and Cvi (lower) seeds.

Histone modifications often occur in combinatorial patterns which establish diverse epigenetic environments influencing gene expression. To investigate such patterns in seeds, we performed a genome-wide correlation analysis of histone methylation marks (H3K4me3, H3K9me3, and H3K27me3) across the three seed states (FH, AR, and GS) in both Col and Cvi. A consistent positive correlation was observed between H3K4me3 and H3K9me3 across all seed states in both Col and Cvi, whereas H3K4me3 showed no significant correlation with H3K27me3 (**Figure 2A**). Notably, H3K9me3 also displayed a weak correlation with H3K27me3. To further analyze combinatorial histone mark depositions at the gene level, we categorized genes by the presence of MACS2-defined peaks for H3K4me3, H3K9me3, and H3K27me3 at their loci (**Supplementary Dataset S1, S2**). Across all seed states, the most abundant category consisted of genes marked with both H3K4me3 and H3K9me3, followed by those marked only with H3K4me3 (**Figure 2B**). Most of the H3K9me3 peaks were colocalized with H3K4me3. By contrast, H3K27me3 was not frequently observed in conjunction with H3K4me3 or H3K9me3. Notably in both Col and Cvi, the proportion of genes marked with both H3K4me3 and H3K9me3 increased from AR to GS, while the proportion of genes marked with only H3K4me3 declined. Heatmaps of H3K4me3, H3K9me3, and H3K27me3 enrichments centered on H3K4me3 or H3K9me3 peaks also revealed similar deposition patterns between H3K4me3 and H3K9me3, whereas H3K27me3 displayed a distinct and somewhat inverse enrichment pattern (**Supplementary Figure 2**). These findings imply a functional interplay between H3K4me3 and H3K9me3 in modulating chromatin structure and gene expression, while highlighting the notion that H3K27me3 plays a functionally and structurally divergent role from H3K4me3 and H3K9me3 marks.

Because a weak genome-wide correlation between H3K9me3 and H3K27me3 was observed (**Figure 2A**), we then specifically examined H3K9me3 enrichment within genes co-marked with H3K27me3 but lacking H3K4me3 (**Figure 2C**). Notably, H3K9me3 levels were relatively low in these genes compared to genes co-marked with H3K4me3 alone. Interestingly, H3K9me3 signals in genes co-marked solely with H3K27me3, displayed a broad distribution resembling that of H3K27me3, whereas in genes co-marked only with H3K4me3, H3K9me3 was sharply localized near TSSs. These findings raise the possibility of partial cross-reactivity of the H3K9me3 antibody used, as have commercial anti-H3K9me3 antibodies been reported to weakly bind H3K27me3 due to a high sequence similarity flanking the modification sites (Egelhofer et al., 2011; Hattori et al., 2013). Nevertheless, a potential for H3K9me3 to exert a functional role also within repressive chromatin cannot be fully excluded. Further experimental validation including antibody-specificity tests and the use of antibodies with higher specificity (for example, clasping antibodies (Riso et al., 2025)) will be required to flawlessly determine the localization and functional relevance of H3K9me3 within repressive chromatin. Even with this limitation, the robust co-enrichment of H3K9me3 with H3K4me3 within H3K27me3-free chromatin clearly demonstrates that H3K9me3 is mainly deposited with active chromatin in Arabidopsis seeds.

### Influence of histone methylation on transcriptional output in FH, AR, and GS seeds

**Figure 3:**
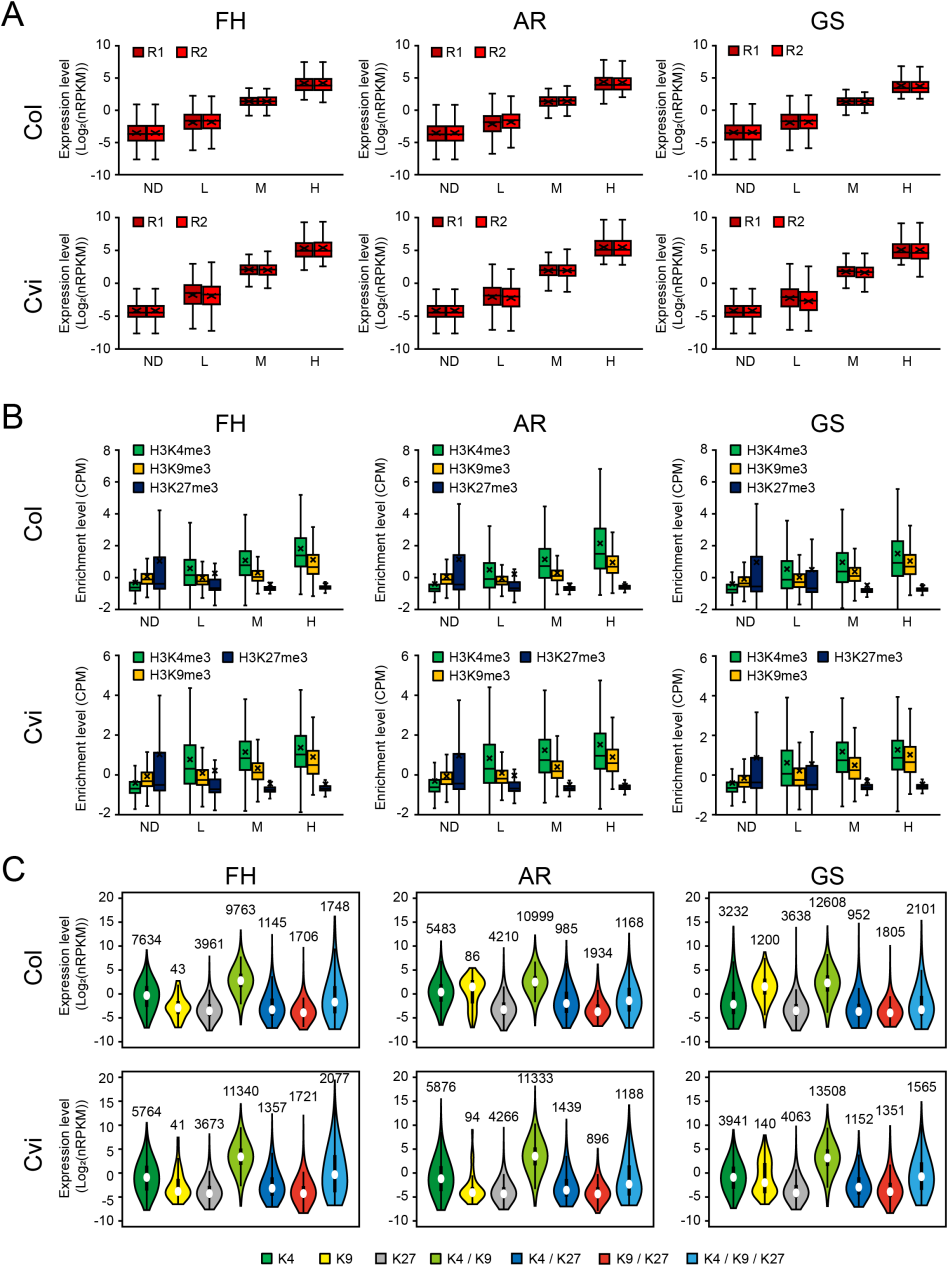
Relationship between gene expression and histone methylations. **(A)** Boxplots showing transcript abundance of genes stratified by expression levels. Expression levels were calculated as normalized RPKM values (see Materials and Methods). H, M, L, and ND indicate genes in the top 33%, middle 33%, lowest 33%, and those with no detectable transcript reads, respectively. Values from two biological replicates (R1 and R2) are indicated. **(B)** Box plots showing the enrichment levels of H3K4me3, H3K9me3, and H3K27me3 across the four gene groups defined in **(A)**. See **Figure 1D** legend for box plot explanation **(A, B)**. **(C)** Violin plots showing the expression levels of genes carrying individual or combinatorial histone marks as defined in **Figure 2B**. The number of genes in each category is indicated above the corresponding plot. Dots represent mean values; boxes indicate interquartile range (25th–75th percentile); whiskers extend to adjacent values. Average values from two biological replicates are shown.

To study the relationship between histone methylation and gene expression, we performed the integrative analysis of ChIP-seq and RNA-seq datasets across FH, AR, and GS states in Col and Cvi seeds. RNA-seq data used for this analysis were previously generated from seed samples prepared under the same physiological conditions as those for the ChIP-seq samples (Lee et al., 2023). All genes were binned into four groups based on their expression levels (**Figure 3A**), and the enrichment of H3K4me3, H3K9me3, and H3K27me3 within each group was quantified (**Figure 3B**). Consistent with previous studies (Roudier et al., 2011; Zhu et al., 2017), H3K4me3 levels were positively correlated with gene expression levels, while H3K27me3 levels were inversely correlated across all seed states in both Col and Cvi seeds. Notably, H3K9me3 levels were also positively correlated with gene expression levels. Together with its predominant localization in euchromatic regions and enrichment around TSSs, this finding suggests that H3K9me3 in plants may play a distinct role from its canonical repressive role in animals. Instead, H3K9me3 may function in a transcriptionally permissive or supportive manner, similar to H3K4me3. Alternatively, it may act as a buffering mechanism to fine-tune or limit excessive transcription.

Next, in order to investigate the combinatorial effects of histone methylation marks on gene expression, we evaluated the expression levels of genes marked with individual histone modification (H3K4me3, H3K9me3, or H3K27me3) or combinations of these marks, including H3K4me3 and H3K9me3 (H3K4me3/H3K9me3), H3K4me3/H3K27me3, H3K9me3/H3K27me3, and H3K4me3/H3K9me3/H3K27me3 (**Figure 3C**). Remarkably, genes marked with H3K4me3/H3K9me3 exhibited significantly higher transcript levels than those marked with either modification alone across the FH, AR, and GS states in Col and Cvi seeds. This observation further supports a potential synergistic interaction between H3K4me3 and H3K9me3 in promoting transcriptional output. Conversely, genes marked with H3K4me3/H3K27me3 or H3K9me3/H3K27me3 displayed lower transcript levels compared to those marked with only H3K4me3 or H3K9me3, respectively, consistent with the repressive role of H3K27me3. These results indicate that H3K4me3 and H3K9me3 are associated with transcriptional activation and may act synergistically, whereas the presence of H3K27me3 in combination with these marks counteracts their activating effects, thereby contributing to the fine-tuning of gene expression in seeds.

### Dynamics of histone methylation during physiological and developmental transitions in seed states

**Figure 4:**
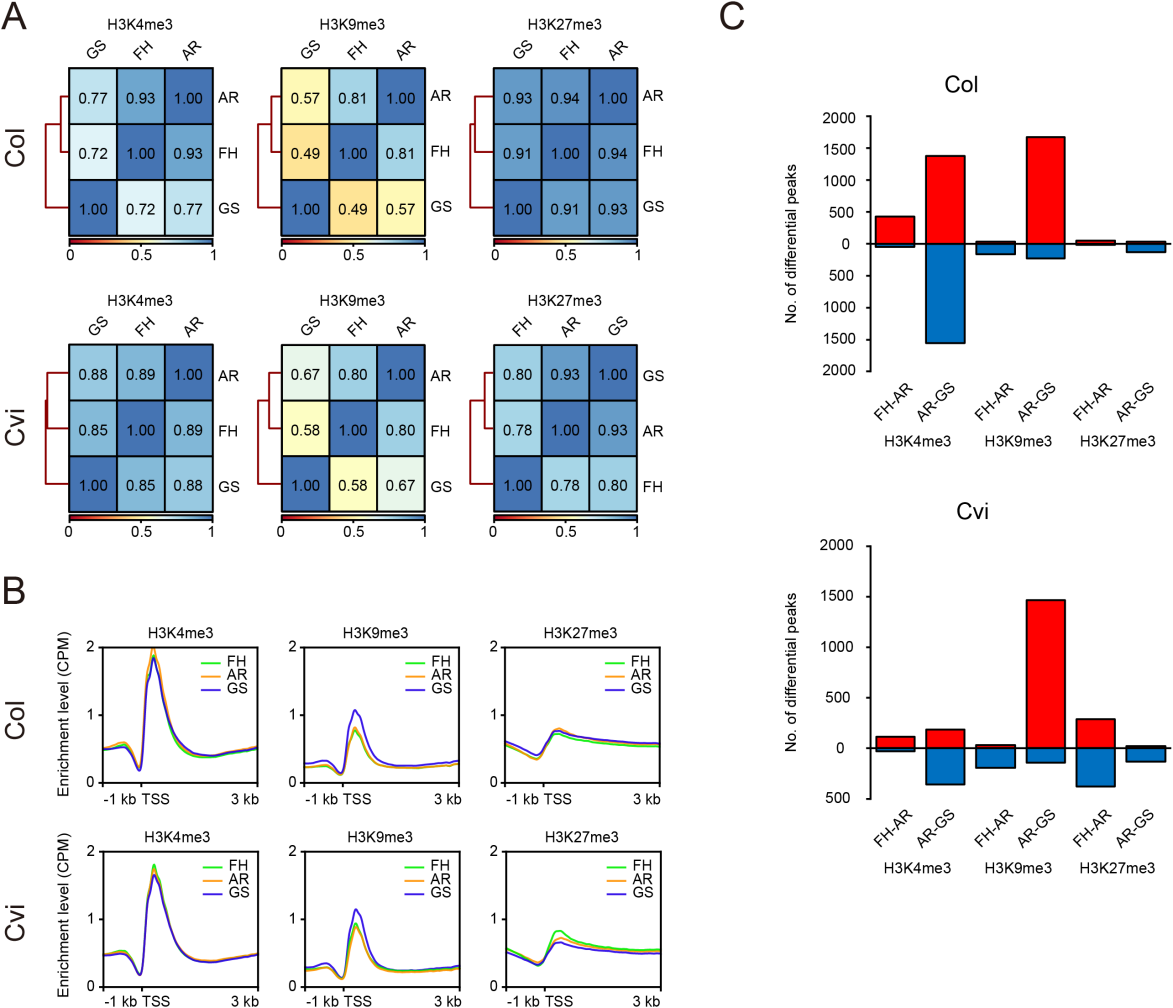
Dynamics of histone methylation during seed-state transitions. **(A)** Heatmaps showing Spearman’s rank correlations of H3K4me3, H3K9me3 and H3K27me3 enrichments across FH, AR, and GS seed states. Correlation coefficients were calculated using CPM values over combined histone methylation peaks from FH, AR, and GS datasets (see Materials and Methods). **(B)** Metaplots showing H3K4me3, H3K9me3, and H3K27me3 enrichments at the FH, AR, and GS states around TSS of all genes. **(C)** Numbers of differential peaks for H3K4me3, H3K9me3, and H3K27me3 during seed-state transitions. DMRs with increased or decreased enrichments during the seed-state transitions are indicated in red and blue, relatively.

Next, to investigate whether genome-wide changes in histone methylation occur during the seed-state transitions, we performed correlation analyses of histone methylation enrichment within peak regions across FH, AR, and GS stages (**Figure 4A**). In Col, strong correlations in histone methylation levels were observed between FH and AR across all marks, with H3K9me3 slightly lower than H3K4me3 and H3K27me3. In contrast, the correlation between GS and the earlier seed states (FH or AR) declined for H3K4me3 and more noticeably for H3K9me3, suggesting a reorganization of active chromatin features during germination. Conversely, H3K27me3 retained a relatively strong correlation across all seed states, indicating greater stability of this repressive mark from dormant state to germination. In Cvi, the overall correlation patterns were similar to those in Col with slight divergences that may reflect potential accession-specific differences. Specifically, the correlations of H3K4me3 and H3K9me3 between AR and GS were slightly higher in Cvi compared to Col, whereas the correlation of H3K27me3 between FH and AR was slightly reduced in Cvi.

Metaplots of histone methylation enrichment in regions surrounding TSSs revealed that H3K4me3 and H3K27me3 levels remained relatively constant across seed states in both Col and Cvi (**Figure 4B**), consistent with the results of the above genome-wide correlation analysis. In contrast, H3K9me3 exhibited more dynamic behavior; its enrichment levels were maintained during the FH-to-AR transition, however showed substantial increases during the AR-to-GS transition in both accessions. This suggests that increase in H3K9me3 might be a conserved epigenetic feature associated with the initiation of germination in Arabidopsis.

To further investigate the dynamics of histone methylation during seed-state transitions, we identified differential peaks for H3K4me3, H3K9me3, and H3K27me3 between FH and AR, and between AR and GS states. We then annotated these differential peaks to nearby genes, designating the resulting gene sets as differentially methylated genes (DMGs) (**Supplementary Dataset S3**, **S4**). The validity of differential peaks was confirmed by ChIP-qPCR of the representative regions showing differential enrichment across transitions (for example, FH > AR, FH < AR, AR > GS, and AR < GS) (**Supplementary Figure 3**, **S4**). Consistent with the correlation and metaplot analyses, the number of differential peaks for H3K4me3 and H3K9me3 was relatively limited during the FH-to-AR transition but markedly increased during the AR-to-GS transition in both Col and Cvi (**Figure 4C**). Remarkably, during the AR-to-GS transition, the number of regions showing increased H3K9me3 substantially outnumbered those with decreased H3K9me3, showing ~ 7 and ~10 times higher in Col and Cvi, respectively (**Figures 4C**, **Supplementary Dataset S3**, **S4**). This suggests a potential crucial role of H3K9me3 in transcriptional activation associated with germination. In line with the correlation analysis (**Figure 4A**), we observed a higher number of differential H3K27me3 peaks between FH and AR in Cvi than in Col, while the number of differential H3K4me3 peaks between AR and GS was lower in Cvi compared to Col. These findings indicate potential accession-specific differences in histone methylation dynamics. Nevertheless, the relatively small number of differential H3K27me3 peaks between AR and GS suggests that H3K27me3-mediated transcriptional repression might play a minor role during germination.

In summary, genome-wide histone methylation landscapes remained largely stable during the FH-to-AR transition, indicating epigenetic continuity during dormancy release. However, the AR-to-GS transition was marked by a substantial increase in H3K9me3 at numerous genomic regions. This pattern suggests that while chromatin states are maintained during dormancy release, H3K9me3 may play a prominent role in facilitating transcriptional reprogramming during germination.

### Conservation of differential histone methylation and dynamics between Col and Cvi

Our analyses revealed that the dynamics of histone methylation during the seed-state transitions were largely similar between Col and Cvi. To investigate whether differential histone methylation is conserved between Col and Cvi seeds, we compared the sets of DMGs associated with the differential H3K4me3 or H3K9me3 peaks in Col and Cvi (**Supplementary Figure 5**). The number of DMGs shared between the two accessions during the FH-to-AR transition was low. Likewise, the overlap of DMGs during the AR-to-GS transition was also lower than expected, despite the broadly similar patterns of H3K4me3 and H3K9me3 dynamics observed in the two accessions. One possible explanation for this limited overlap would be that the stringent criteria used for differential-peak calling might have excluded regions containing small yet significant changes in methylation. To address this possibility, we extended our analysis by assessing H3K4me3 and H3K9me3 levels in Cvi across the differential peaks identified in Col. In a reciprocal manner, histone methylation levels in Col were assessed across the differential peaks identified in Cvi. Notably, differential peaks identified in Col showed statistically significant differences in H3K4me3 and H3K9me3 levels in Cvi also, and *vice versa* (**Supplementary Figure 6**). These results show that, although individual differential peaks may differ potentially due to peak-calling thresholds, the corresponding genomic regions undergo comparable changes in H3K4me3 and H3K9me3 enrichment in both Col and Cvi. Although genome-wide changes in histone methylation were limited (particularly during the FH-to-AR transition), the presence of regions with consistent histone methylation changes across accessions suggests that localized epigenetic modifications may contribute to gene regulation and chromatin remodeling during seed-state transitions. Despite being limited in number, such regions may serve as regulatory hotspots with functional relevance in the control of dormancy- or germination-associated gene expression.

### Relationship between histone methylation dynamics and transcriptional output during seed-state transitions

**Figure 5:**
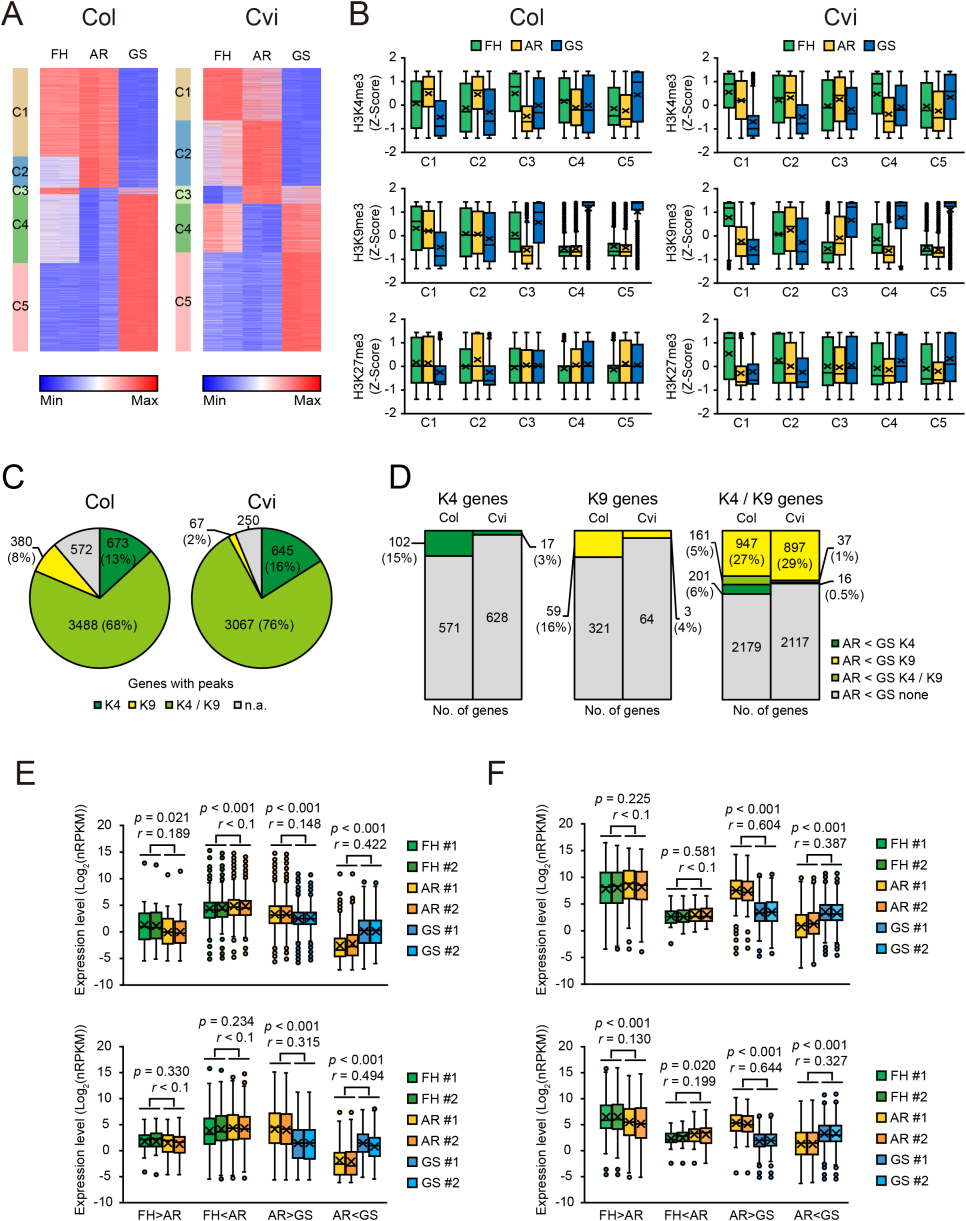
Relationship between histone methylation dynamics and transcriptomic changes during seed-state transitions. **(A)** Heatmaps showing *k*-means clustering (*k* = 5) of DEGs identified from FH vs. AR and AR vs. GS comparisons, based on their expression profiles across FH, AR, and GS states in Col and Cvi. Expression levels from two biological replicates are shown. See text for gene clusters C1 to C5. **(B)** Z-scored histone methylation signals for H3K4me3, H3K9me3 and H3K27me3 across FH, AR, and GS states in Col and Cvi for gene clusters C1 to C5. See **Figure 1D** legend for box plot explanation. **(C)** Pie charts showing the proportions of genes in clusters C4 and C5 carrying histone methylation peaks at the GS state. K4 and K9 indicate genes marked only with H3K4me3 and H3K9me3, respectively; K4/K9 indicates genes marked with both H3K4me3 and H3K9me3; n.a. denotes genes lacking both marks. **(D)** Bar plots showing the numbers and proportions of genes in clusters C4 and C5 harboring AR < GS differential peaks for H3K4me3 (AR < GS K4), H3K9me3 (AR < GS K9), both marks (AR < GS K4/K9), or neither mark (AR < GS none) at the GS state. **(E, F)** Expression levels of DMGs for H3K4me3 (upper) and H3K9me3 (lower) in Col **(E)** and Cvi **(F)**. Values from two biological replicates (#1 and #2) are indicated. Statistical significance was evaluated using the two-tailed Mann-Whitney U test; *p* refers to the adjusted *p*-value, and *r* denotes the effect size. See **Figure 1D** legend for box plot explanation.

The analysis of histone methylation dynamics suggested that targeted, rather than global, changes in histone methylation may affect transcription during seed-state transitions. To further explore this, we examined whether histone methylation dynamics are associated with transcriptome changes in the three seed states. Differentially expressed genes (DEGs) were identified for the FH-to-AR and AR-to-GS transitions (**Supplementary Dataset S5, S6**). Notably, the number of DEGs was substantially higher during the AR-to-GS transition than the FH-to-AR transition (**Supplementary Figure 7**), in line with the pattern observed for differential H3K4me3 and H3K9me3 peaks (**Figure 4C**). Next, all DEGs were pooled and divided into five clusters (C1 to C5; **Figure 5A**) by applying *k*-means clustering. Gene Ontology (GO) analysis revealed that genes within clusters C1 and C2 of Col and Cvi, which are downregulated during the AR-to-GS transition, were enriched for GO terms associated with stress-related processes such as water deprivation, hypoxia, and ABA signaling (**Supplementary Figure 8**, **Supplementary Dataset S7**). On the other hand, genes in clusters C4 and C5, which are upregulated during the AR-to-GS transition, were enriched for GO terms related to translation, fatty-acid biosynthesis, cell division, differentiation, developmental growth, and cellular component biogenesis (**Supplementary Figure 8**, **Supplementary Dataset S7**).

To determine the relationship between histone methylation and gene expression across these clusters, we quantified the levels of H3K4me3, H3K9me3, and H3K27me3 at the genes within these clusters in all seed states and presented the values as row Z-scores (**Figure 5B**, **Supplementary Dataset S8**). Overall, the patterns of expression dynamics generally correlated with the changes in H3K4me3 and H3K9me3 levels in both Col and Cvi. Remarkably, substantial correlations were observed between H3K9me3 dynamics and transcriptome dynamics for clusters C4 and C5 of Col and Cvi, where genes were relatively lowly expressed at FH and AR states but activated during the AR-to-GS transition. In comparison, H3K4me3 showed a more modest correlation with gene expression changes than H3K9me3 during this transition.

To further explore the role of histone methylation in regulating clusters C4 and C5, we examined H3K4me3 and H3K9me3 depositions for genes in clusters C4 and C5 at the GS state. The largest proportion of the genes—68% in Col and 76% in Cvi—were marked with both H3K4me3 and H3K9me3 (**Figure 5C**). Among these, 27% (Col) and 29% (Cvi) contained AR < GS differential H3K9me3 peaks, while only 6% and 0.5%, respectively, had AR < GS differential H3K4me3 peaks, and 5% and 1%, respectively, harbored differential peaks for both marks (**Figure 5D**). On the other hand, 13% (Col) and 16% (Cvi) of the genes were marked with H3K4me3 alone (**Figure 5C**), of which 15% and 3%, respectively, associated with AR < GS differential H3K4me3 peaks (**Figure 5D**). Genes marked solely with H3K9me3 were relatively rare (8% in Col and 2% in Cvi), and among them, 16% (Col) and 4% (Cvi) showed AR < GS differential H3K9me3 peaks (**Figure 5D**).

A comparison of DEGs and DMGs further supported the findings above (**Supplementary Figure 9**). Notably, ~20% of the genes upregulated during the AR-to-GS transition contained AR < GS differential H3K9me3 peaks in both accessions, whereas only ~9% (Col) and ~2% (Cvi) were associated with AR < GS differential H3K4me3 peaks. Moreover, transcript levels of the genes marked with differential H3K4me3 or H3K9me3 peaks during the AR-to-GS transition correlated positively with H3K4me3 and H3K9me3 levels in both accessions (**Figures 5E, F**). In contrast, DMGs identified during the FH-to-AR transition displayed relatively constant or only slightly changed transcript levels (**Figures 5E, F**). Taken together, these results further support the idea that H3K9me3 might act as a key epigenetic regulator modulating transcriptional reprogramming during the AR-to-GS transition and potentially promoting the expression of genes essential for germination.

### Relationship between histone methylation and transcription initiation during the AR-to-GS transition

It has been generally believed that transcriptional activity in dry seeds is largely suppressed due to chromatin compaction and dehydration occurring during seed maturation. The relatively weak correlation between transcriptome dynamics and histone methylation during the FH-to-AR transition may reflect the transcriptional quiescence status of dry seeds. However, a recent capped-small RNA sequencing (csRNA-seq) of dry seeds by Tremblay et al (Tremblay et al., 2024). detected nascent capped transcripts in dry seeds, raising the possibility that transcription initiation may occur in dry seeds, although it remains unclear whether these RNA polymerase-bound transcripts undergo productive elongation. Tremblay et al (Tremblay et al., 2024). also demonstrated that germinating seeds contain *de novo* synthesized transcripts in addition to stored RNAs.

To examine whether H3K4me3 and H3K9me3 are associated with potential transcription initiation, we identified differentially expressed transcription start sites (DE TSSs) between DS (dry seeds) and L26 (26-hour light-imbibed seeds) states (Tremblay et al., 2024) that were comparable to the AR and GS states of this study (**Supplementary Figure 10** and **Supplementary Dataset S9**). Genes in each cluster were classified based on whether they overlapped with DE TSS-associated genes. Genes in clusters C1 and C2 overlapped with DS > L26 DE TSSs (i.e., downregulated during germination), whereas genes in clusters C3, C4, and C5 overlapped with DS < L26 DE TSSs (i.e., upregulated during germination). Notably, increases in H3K9me3 levels during the AR-to-GS transition were more pronounced in C3, C4, and C5 cluster genes showing overlap with DE TSSs than genes showing no overlap (**Supplementary Figure 10**). Interestingly, reductions in H3K9me3 levels, and to a lesser extent, H3K4me3 levels, during the AR-to-GS transition were also more evident in C1 and C2 cluster genes showing overlap with DE TSSs than genes showing no overlap (**Supplementary Figure 10**). These results indicate that changes in H3K4me3 and particularly H3K9me3 levels are associated with transcriptional initiation events as captured by csRNA-seq during germination.

### Biological functions of genes enriched with H3K9me3 during germination

**Figure 6:**
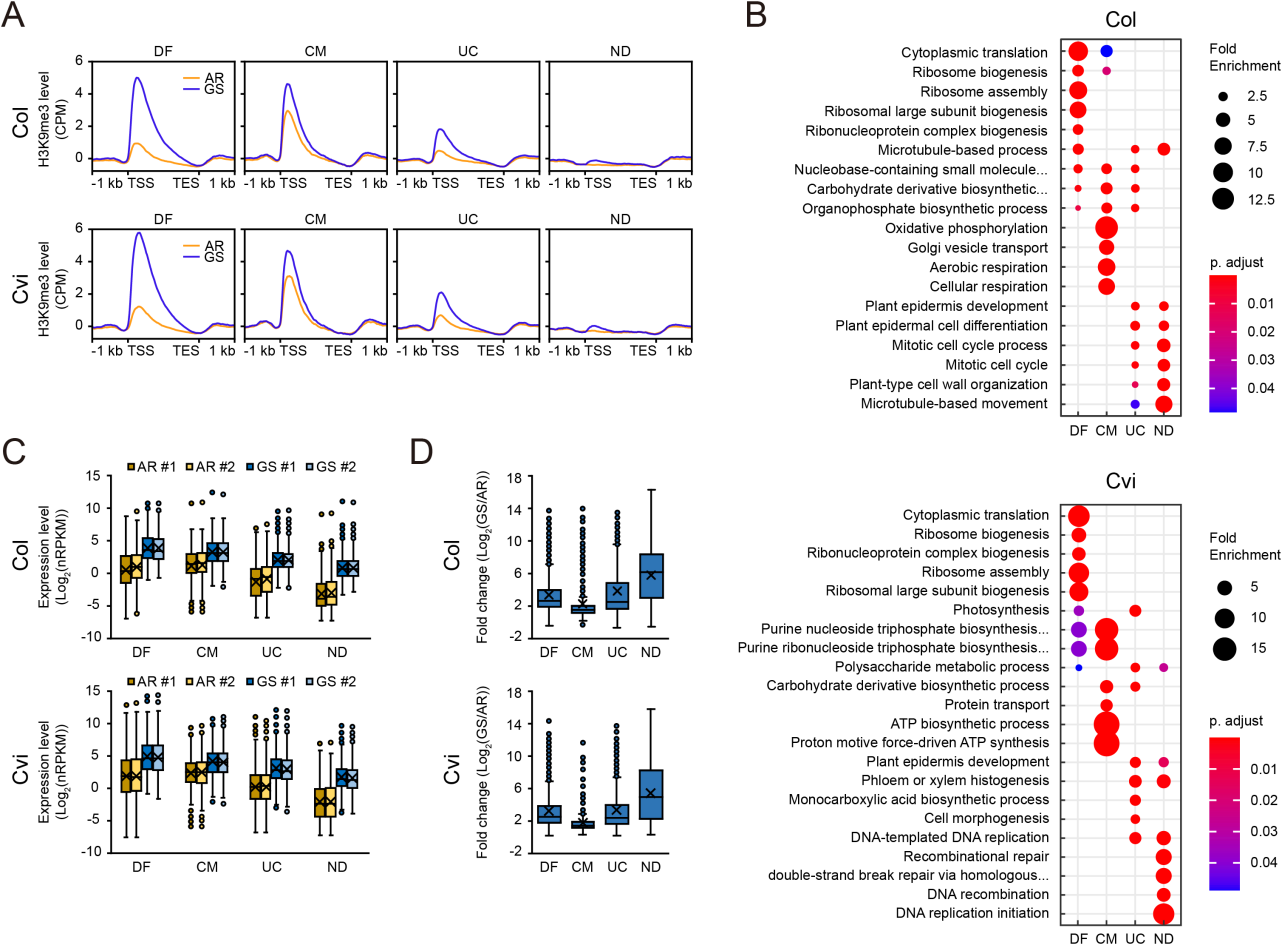
Characteristics of genes in clusters C4 and C5 categorized by H3K9me3 status during the AR to GS transition. **(A)** Metaplots showing H3K9me3 enrichment at the AR and GS states for genes in clusters C4 and C5 in Col and Cvi. Categories: DF (differential AR < GS peaks), CM (common peaks in AR and GS), UC (uncategorized peaks), and ND (no detectable peaks). **(B)** Dot plots showing enriched GO terms (biological processes; FDR < 0.05) for each category defined in **(A)**. Dot size indicates the fold enrichment of genes associated with each GO term, and color reflects the FDR value for enrichment. **(C, D)** Box plots showing expression levels at the AR and GS states **(C)**, and fold change in expression during the AR-to-GS transition **(D)** for each category defined in **(A)**. Values from two biological replicates (#1 and #2) are indicated in **(C)**, whereas average values from two biological replicates are shown in **(D)**. See **Figure 1D** legend for box plot explanation.

A substantial portion of GS-upregulated genes carrying AR < GS differential H3K9me3 peaks led us to investigate whether these genes possess distinct functional characteristics compared to the rest of GS-upregulated genes. To address this, we classified genes in clusters C4 and C5 into four groups: (1) genes with AR < GS differential H3K9me3 peaks, (2) genes with common H3K9me3 peaks between AR and GS, (3) genes with H3K9me3 peaks that were neither classified as AR < GS differential H3K9me3 peaks nor as common peaks, and (4) genes lacking detectable H3K9me3 peaks (**Figure 6A** and **Supplementary Table 2**).

To elucidate the functional relevance of these groups, we performed GO analyses for each of the four groups (**Figure 6B** and **Supplementary Dataset S10**). Notably, enriched GO terms varied among the groups, suggesting potential functional divergence. In Col, genes with AR < GS differential H3K9me3 peaks were primarily associated with GO terms such as translation and ribosome biogenesis, suggesting their roles in ensuring translation capability of germinating seeds. In contrast, genes with non-differential (common) H3K9me3 peaks were enriched with GO terms related to energy metabolism such as oxidative phosphorylation, aerobic respiration, and ATP biosynthesis, along with comparatively modest enrichment for GO terms related to translation. The third group exhibited enrichment for cellular/tissue differentiation and carbohydrate metabolism. Although this group displayed relatively low H3K9me3 levels, yet the levels increased more than two-fold during the AR-to-GS transition (**Figure 6A**). Finally, the fourth group showed a strong enrichment for GO terms related to cell cycle. Furthermore, the GO terms enriched among GS-upregulated genes with AR < GS differential H3K4me3 peaks differed from those associated with AR < GS differential H3K9me3 peaks (**Supplementary Figure 11**).

Genes carrying AR < GS differential H3K9me3 peaks in Cvi were also predominantly enriched for translation-related GO terms, resembling the trend observed in Col (**Figure 6B**). Genes with common H3K9me3 peaks (the second group) exhibited enrichment for protein glycosylation, ATP metabolic processes, and biosynthetic processes of cellular macromolecules. The third group showed enrichment for cellular/tissue differentiation and diverse biosynthetic and metabolic processes. Similar to the observation in Col, genes lacking H3K9me3 peaks in Cvi (the fourth group) were enriched with cell cycle and DNA-replication-related processes. These results show that functionally diverse genes are subjected to different epigenetic regulation during germination.

To address the potential regulatory role of H3K9me3 during germination, we also analyzed gene expression levels and fold changes during the AR-to-GS transition across the four groups (**Supplementary Dataset S11**). In all groups, transcript levels at the AR and GS states positively correlated with H3K9me3 levels (**Figure 6C**), supporting a positive correlation between gene expression and H3K9me3 levels. Fold changes in gene expression were also positively correlated with changes in H3K9me3 enrichment, except for genes lacking H3K9me3 peaks (**Figure 6D**). Genes in the first and third groups exhibited greater fold induction than the genes in the second group. Genes lacking H3K9me3 peaks (the fourth group) showed the highest and most variable fold inductions. These correlative patterns were commonly observed in Col and Cvi. Taken together, the results above indicate that H3K9me3 is enriched during germination selectively at genes that require high expression levels, especially those involved in translation.

### Changes in histone methylation and transcriptional output during air-ripening

**Figure 7:**
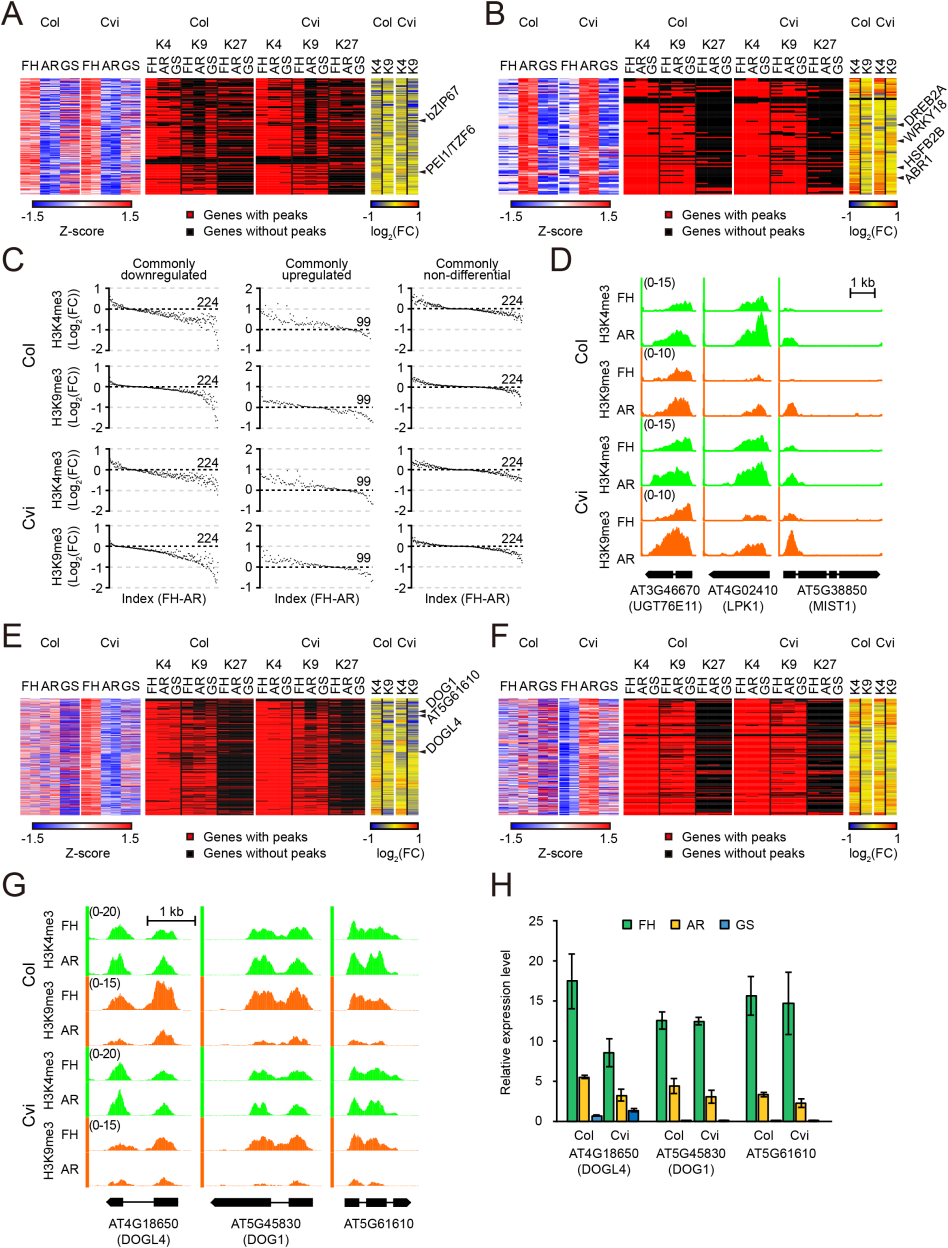
Relationship between transcript and histone methylation changes during air-ripening. **(A, B)** Left panels: Heatmaps showing row Z-scores of normalized RPKM values for hierarchically clustered genes that are down-regulated **(A)** or up-regulated **(B)** during the FH to AR transition in both Col and Cvi. Results from two biological replicates are shown. Middle panels: Binary maps showing presence (red) or absence (black) of H3K4me3 (K4), H3K9me3 (K9), or H3K27me3 (K27) mark for each gene. Right panels: Heatmaps showing log_2_ fold changes in H3K4me3 (K4) or H3K9me3 (K9) levels between FH and AR calculated as log_2_(AR + 1/FH+1). **(C)** Ranked scatter plots showing log_2_ fold change in H3K4me3 or H3K9me3 level per gene, with genes ordered by the difference in histone methylation levels between FH and AR. **(D)** Genome browser snapshots showing H3K4me3 or H3K9me3 ChIP-seq signal tracks at representative gene loci in the FH and AR seeds of Col and Cvi. **(E, F)** Left panels: Heatmaps displaying row Z-scores of normalized RPKM values for hierarchically clustered genes that are downregulated **(E)** or upregulated **(F)** specifically in Cvi during the FH-to-AR transition. Results from two biological replicates are shown. Middle panels: Binary maps indicating the presence (red) or absence (black) of H3K4me3 (K4), H3K9me3 (K9), or H3K27me3 (K27) mark. Right panels: Heatmaps showing log_2_ fold changes in H3K4me3 (K4) or H3K9me3 (K9) levels between FH and AR calculated as log_2_(AR + 1/FH+1). **(G)** Genome browser views of H3K4me3 or H3K9me3 ChIP-seq signal tracks at representative genes selected from **(E)** in Col and Cvi seeds at the FH and AR states. **(H)** Transcript levels of the genes shown in **(G)** as measured by RT-qPCR in Col and Cvi seeds at the FH and AR states. *PP2A* was used as an expression control for normalization. Values represent the mean ± SE from three biological replicates. Each biological replicate was analyzed using three technical replicates.

Although active transcription is unlikely to occur in dry seeds, previous studies have reported transcriptomic differences between seeds at FH and AR states. Consistently, we also identified numerous DEGs during the FH-to-AR transition (**Figure 7**). While the functional relevance of these DEGs to dormancy regulation remains unclear, we sought to identify candidate genes potentially associated with maintenance or release of seed dormancy by analyzing genes upregulated or downregulated during the FH-to-AR transition both in Col and Cvi. Genes with a log_2_(fold change) threshold of ≥ |1.0| in both accessions were selected and further refined by selecting those overlapping with the FH vs. AR DEGs obtained from an independent deep RNA-seq experiment using Cvi FH and AR seeds (see Materials and Methods). We then investigated the histone methylation status of these DEGs to explore the possibility that histone methylation contributes to the regulation of seed dormancy-associated gene regulation during air-ripening (**Figures 7A–D**, **Supplementary Dataset S12**).

In total, 224 and 101 genes were commonly downregulated and upregulated, respectively, in both Col and Cvi during the FH-to-AR transition. The common AR-repressed gene group included those encoding seed storage proteins, enzymes involved in lipid biosynthesis, and proteins with diverse functions (**Supplementary Dataset S13**). Among them, genes encoding transcription factors were of particular interest due to their potential regulatory roles in dormancy-related gene expression. Notably, genes encoding transcription factors linked to dormancy or germination, such as *bZIP67* (*AT3G44460*), a trans-activator of *DOG1* (Bryant et al., 2019), and *PEI1/TZF6* (*AT5G07500*), a negative regulator of germination (Bogamuwa and Jang, 2013), were found in this group (**Figure 7A**, **Supplementary Dataset S13**).

A histone methylation profiling revealed that the majority of the AR-repressed genes were marked with H3K4me3, with a smaller subset with H3K9me3. In contrast, only a relatively small portion of the genes were marked with H3K27me3, suggesting a minor role of H3K27me3 in repressing these genes. At the AR state, a notable reduction in the number of H3K9me3 peaks was observed, suggesting decreased enrichments at many loci to levels below the peak-detection threshold. Comparison of histone methylation levels between FH and AR revealed differential enrichments for a substantial subset of AR-repressed genes in both Col and Cvi (**Figure 7A**). Overall, histone methylation changes were modest, with most genes exhibiting less than two-fold differences and only a small subset showing more pronounced reductions (**Supplementary Table 3**). Specifically, ~43% (Col) and ~ 33% (Cvi) of AR-repressed genes showed a reduction in H3K4me3 (log_2_(AR + 1/FH+1) < -0.3) with a subset, 21% (Col) and 14% (Cvi), showing more substantial reductions (log_2_(AR + 1/FH+1) < -0.5). Decreases in H3K9me3 were also observed in 21% (Col) and 47% (Cvi) of these genes, with a subset, 11% (Col) and 30% (Cvi), showing more pronounced changes (log_2_(AR + 1/FH+1) < -0.5). These results suggest the possibility that subsets of AR-repressed genes may be transcriptionally more active in FH seeds than in AR seeds and that their downregulation during air-ripening may be mediated at least partially by the reduction of activating marks such as H3K4me3 and H3K9me3.

Common AR-upregulated genes, which were fewer in number than AR-downregulated genes, were associated with responses to abiotic and biotic stresses (**Supplementary Dataset S13**). This group contained genes encoding stress-related transcription factors such as *DREB2A* (*AT5G05410*), *ABR1* (*AT5G64750*), *WRKY18* (*AT4G31800*), and *HSFB2B* (*AT4G11660*) etc. (**Figure 7B**, **Supplementary Dataset S13**), suggesting their potential role in safeguarding seed viability during dry storage following dormancy release. Similar to the AR-downregulated genes, the majority of the AR-upregulated genes were marked with H3K4me3 or H3K9me3, but not with H3K27me3 (**Figure 7B**). Histone methylation changes among AR-upregulated genes were less pronounced overall compared to those detected in AR-downregulated genes, especially for H3K9me3 in Col (**Figure 7B**, **Supplementary Table 3**). Approximately, 38% (Col) and 31% (Cvi) of AR-upregulated genes showed increased H3K4me3 levels (log_2_(AR + 1/FH+1) > 0.3), with 18% (Col) and 17% (Cvi) exhibiting larger magnitude increase (log_2_(AR + 1/FH+1) > 0.5). Increases in H3K9me3 were observed in a smaller fraction of genes —7% (Col) and 19% (Cvi)— with a limited subset showing higher magnitude changes (log_2_(AR + 1/FH+1) > 0.5).

Given that active transcription is generally restricted in dry seeds, the modest or insignificant increases in H3K4me3 and H3K9me3 suggest that elevated transcript levels of the AR-upregulated genes may not be the result of *de novo* transcription during air-ripening. Nevertheless, a small number of AR-upregulated genes exhibited clear gains in H3K4me3 or H3K9me3 in both Col and Cvi, raising the possibility of selective transcriptional activation for specific loci during the FH-to-AR transition (**Figures 7C, D**).

To determine whether the observed histone methylation changes were gene-specific or the result of random, biologically irrelevant fluctuation during air-ripening, we analyzed a control set of randomly selected non-differentially expressed genes (non-DEGs) (**Figure 7C**). The vast majority of non-DEGs exhibited stable levels of histone methylation, with no significant skew toward either an increase or decrease in enrichments. These findings indicate that the alterations in H3K4me3 and H3K9me3 levels observed in AR-downregulated genes—and the H3K4me3 changes in AR-upregulated genes—are not attributable to a global shift in histone methylation patterns. These results further support that transcriptional repression during the FH-to-AR transition is associated with the targeted reduction of H3K4me3 and H3K9me3 marks.

We also sought to identify genes that may contribute to variation in dormancy depth by focusing on Cvi-specific transcriptional changes (**Figures 7E–H**). Genes with a log_2_(fold change) threshold of ≥ |1.0| in Cvi but < |0.5| in Col were selected and refined further by comparison using the deep RNA-seq data. This yielded 459 and 277 Cvi-specific AR-downregulated and AR-upregulated genes, respectively (**Supplementary Dataset S12**). Overall patterns of histone methylation mark composition and the differential enrichment of H3K4me3 and H3K9me3 for these genes (**Figures 7E, F**) resembled those of the common AR-regulated genes (**Figures 7A, B**).

Interestingly, Cvi-specific DEGs exhibited a degree of similarity in H3K4me3 and H3K9me3 dynamics in Col during the FH-to-AR transition. For example, the master dormancy regulator *DOG1* (*AT5G45830*) and its homolog *DOGL4* (*AT4G18650*) showed significant decreases in H3K9me3 level in both Col and Cvi (**Figure 7G**), even though RNA-seq data did not indicate differential expression in Col. This discrepancy may be attributed to experimental variation, possibly due to a later harvest of FH seeds used for Col RNA-seq relative to those used for ChIP-seq, which were harvested independently in different laboratories. Supporting this, our RT-qPCR analysis showed that these genes were indeed downregulated in Col AR seeds, consistent with the expression patterns observed in Cvi (**Figure 7H**). Collectively, these results indicate conserved dynamics of chromatin across Col and Cvi at dormancy regulated loci. Our findings indicate that transcriptional shift associated with dormancy release is accompanied by targeted reduction of activating histone marks, specifically H3K4me3 and H3K9me3. The reduction of these marks may cause reduced transcriptional output, or alternatively, may simply reflect a downstream consequence of reduced transcription.

## Conclusions

Our study reveals that H3K9me3, typically regarded as a repressive histone mark, is positively associated with transcription in Arabidopsis seeds and increases markedly during germination. Histone methylation profiles remained largely stable from FH to AR, but dormancy release was accompanied by reductions in H3K4me3 and H3K9me3 at a subset of downregulated genes. These findings suggest a plant-specific role for H3K9me3 as an active or permissive chromatin mark that supports transcriptional reprogramming during germination. More broadly, they highlight potential kingdom-level differences in histone modification function, expanding our understanding of epigenetic diversity across eukaryotes.

## Data Availability

ChIP-seq data have been deposited in the Gene Expression Omnibus (GEO) under the SuperSeries accession number GSE305225. Deep RNA-seq data have been deposited in the GEO under the SuperSeries accession number GSE270762. All other data are available from the authors upon reasonable request.
