# Supplementary material for: Plant-specific function of H3K9me3 as a permissive chromatin mark during Arabidopsis seed germination
Source: Front Plant Sci. 2026 Mar 12;17:1785818. doi: 10.3389/fpls.2026.1785818 (PMC13017857; doi:10.3389/fpls.2026.1785818)
Supplement: Supplementary file 1 [file DataSheet1.pdf]

Supplementary data

Plant-specific function of H3K9me3 as a permissive chromatin mark during Arabidopsis seed germination

Jae-Wook Yoon, Min-Jeong Kang, Hongshi Jin, Yoo-Sun Noh, and Bosl Noh

Supplementary figures

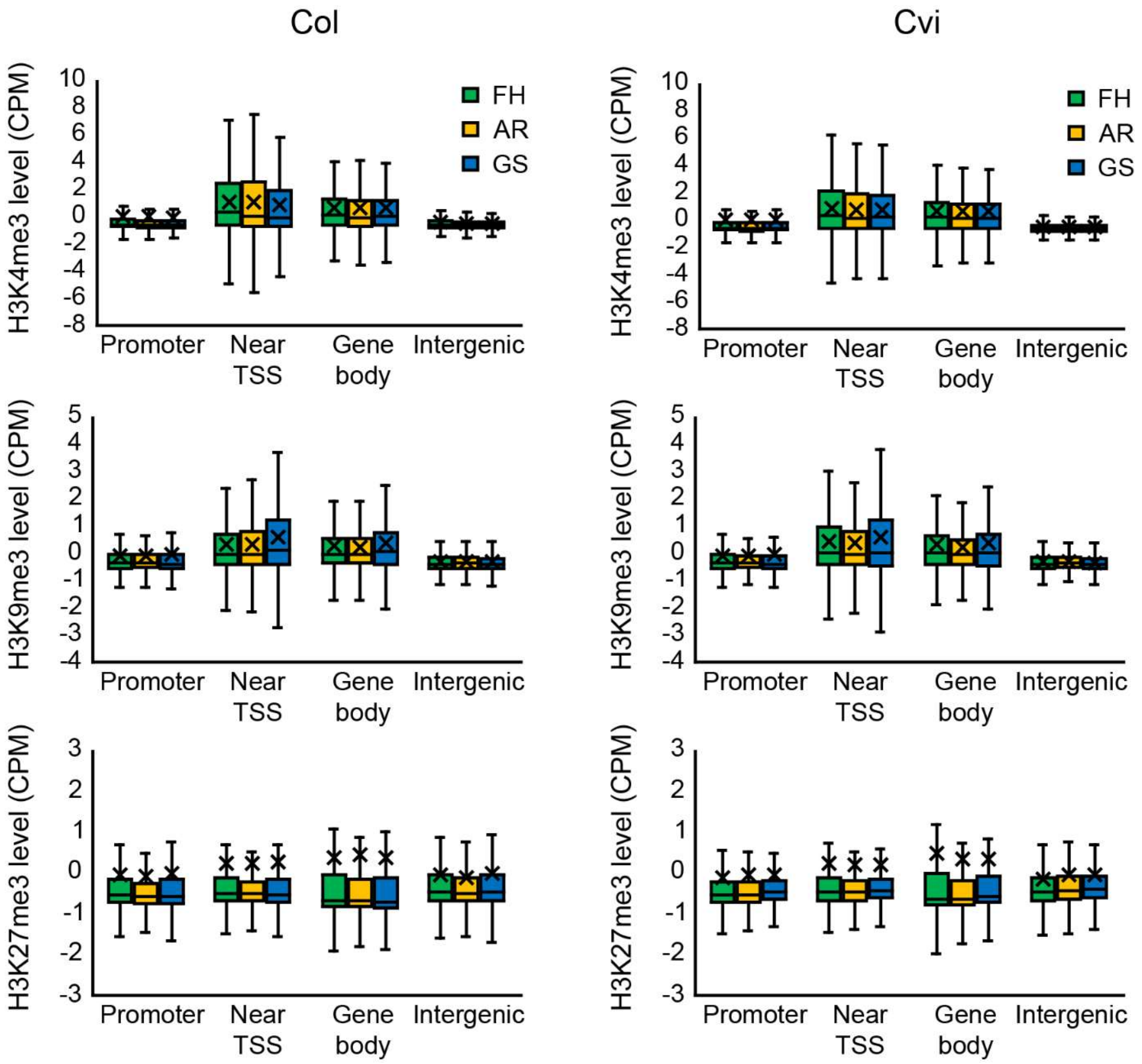

Supplementary Figure S1. H3K4me3, H3K9me3, and H3K27me3 enrichments across genomic features in FH, AR, and GS seeds. Box plots showing the enrichment levels of H3K4me3, H3K9me3, and H3K27me3 across promoter (− 1 kb from TSS), near TSS (+/- 0.3 kb around TSS), gene body (TSS to TES), and intergenic regions. See Figure 1D legend for box plot explanation.

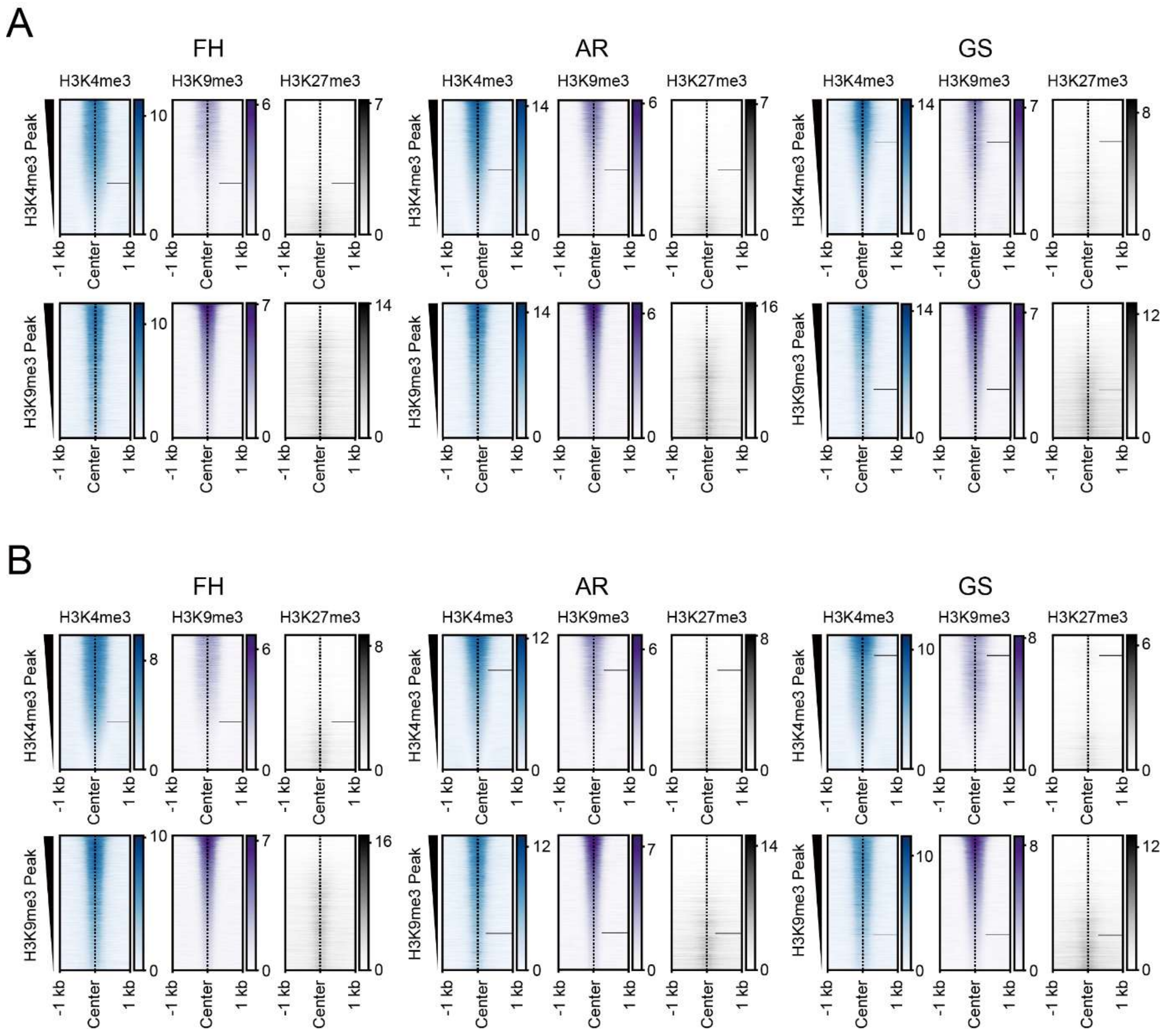

Supplementary Figure S2. Reciprocal enrichments of H3K4me3 and H3K9me3. (**A-B**) Heatmaps showing H3K4me3, H3K9me3, and H3K27me3 signals within ± 1 kb of peak centers for H3K4me3 (upper) and H3K9me3 (lower) at the FH, AR, and GS states in Col (A) and Cvi (B). Peaks are ordered by descending signal intensity of H3K4me3, H3K9me3, and H3K27me3, respectively.

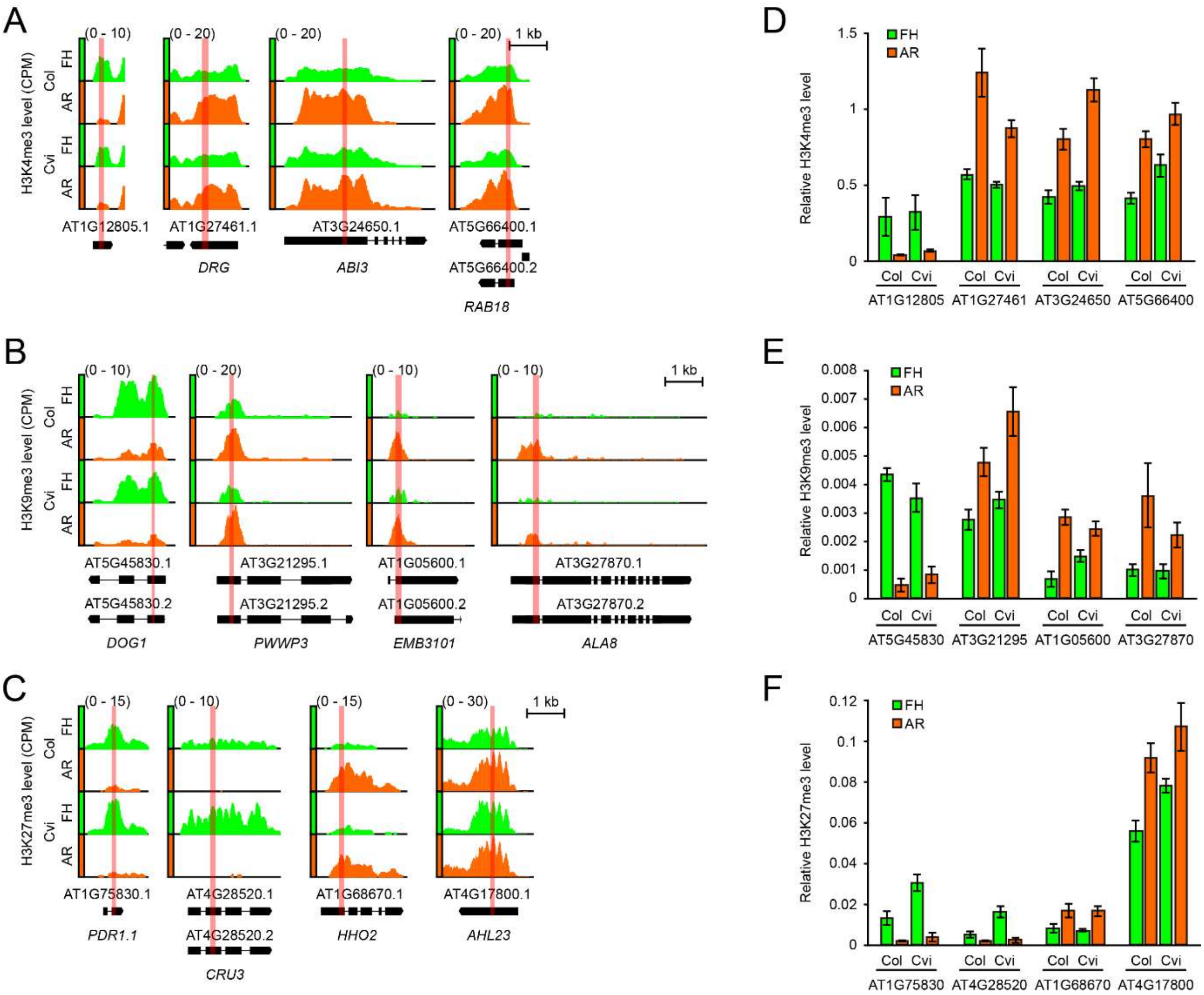

Supplementary Figure S3. ChIP-qPCR validation of histone methylation changes during the FH-to-AR transition identified by ChIP-seq. (**A**-**C**) Genome browser views of H3K4me3 (A), H3K9me3 (B), and H3K27me3 (C) signal tracks at representative gene loci at the FH and AR states. Highlighted regions indicate the sites quantified by ChIP-qPCR in (D-F). (**D**-**F**) ChIP-qPCR analysis showing enrichment of H3K4me3 (D), H3K9me3 (E), and H3K27me3 (F) at the highlighted sites shown in (A-C). Values represent the means ± SE of three biological replicates after normalization to corresponding input controls. Each biological replicate was analyzed using three technical replicates.

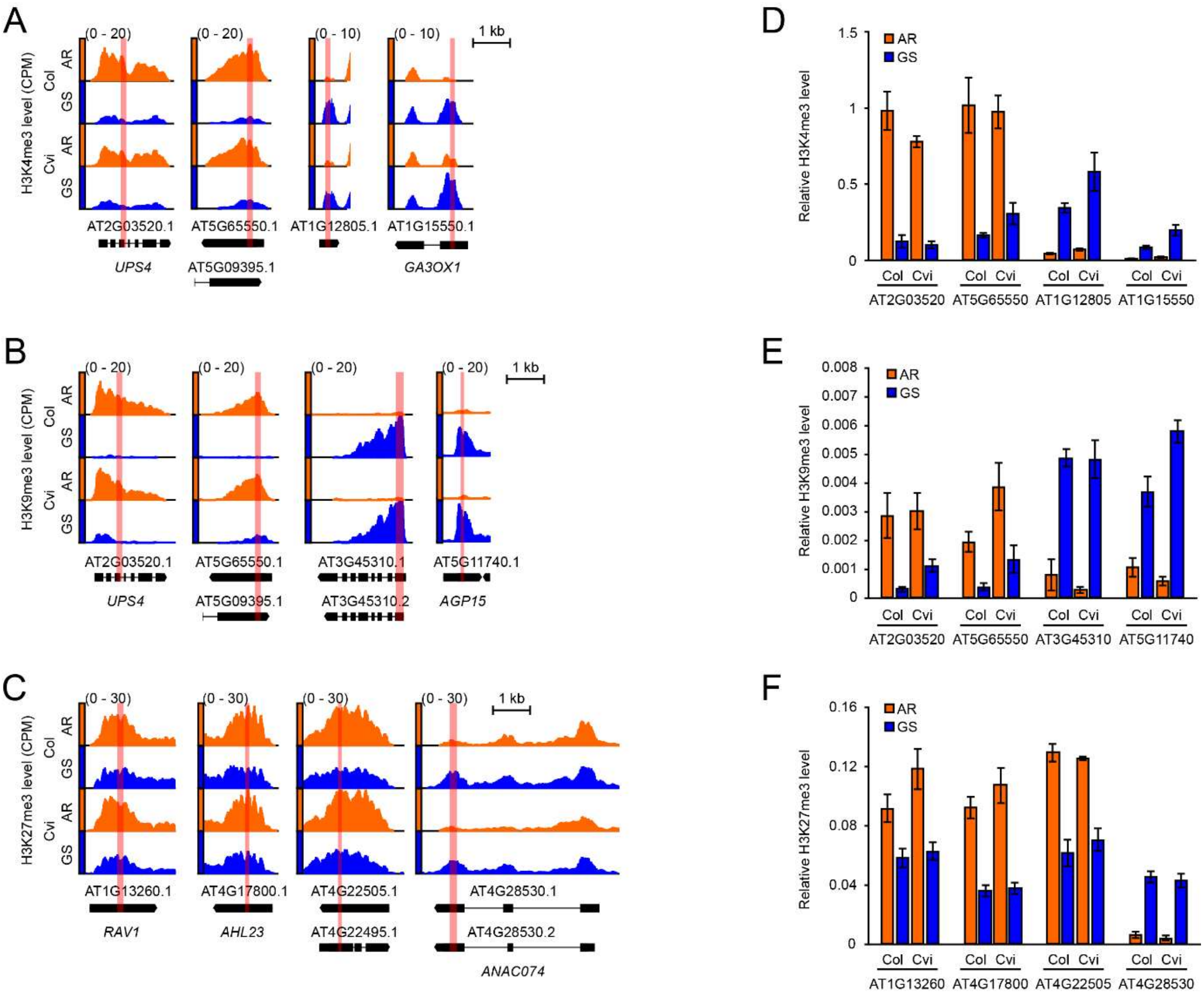

Supplementary Figure S4. ChIP-qPCR validation of histone methylation changes during the AR-to-GS transition identified by ChIP-seq. (**A**-**C**) Genome browser views of H3K4me3 (A), H3K9me3 (B), and H3K27me3 (C) signal tracks at representative gene loci at the AR and GS states. Highlighted regions indicate the sites quantified by ChIP-qPCR in (**D**-**F**). (D-F) ChIP-qPCR analysis showing enrichment of H3K4me3 (D), H3K9me3 (E), and H3K27me3 (F) at the highlighted sites shown in (A-C). Values represent the means ± SE of three biological replicates after normalization to corresponding input controls. Each biological replicate was analyzed using three technical replicates.

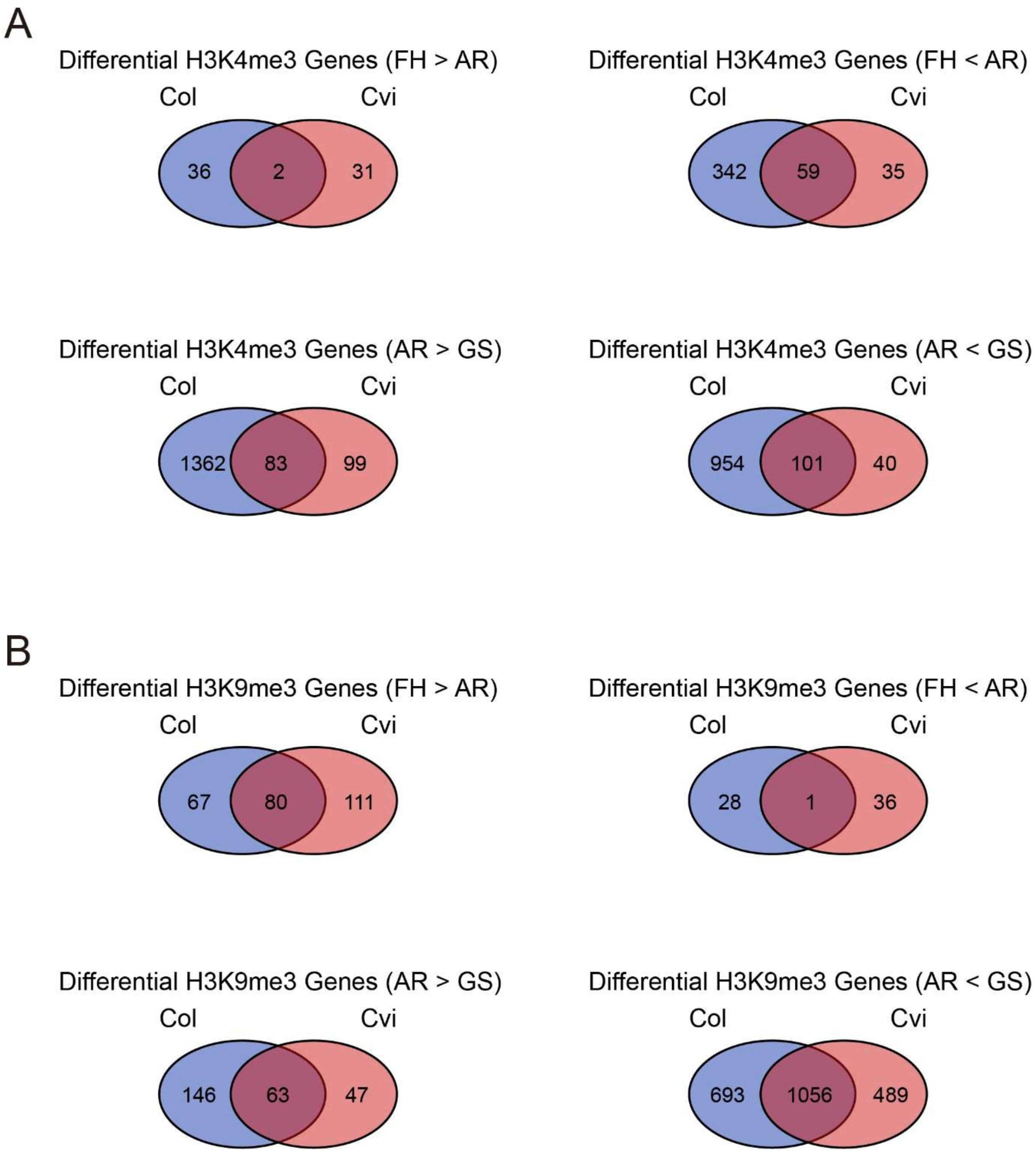

Supplementary Figure S5. Overlap analysis of differentially methylated genes (DMGs) identified in Col and Cvi. (**A-B**) Venn diagrams showing the overlap between H3K4me3 (A) and H3K9me3 (B) DMGs in Col and Cvi that exhibit changes in the same direction during seed transitions.

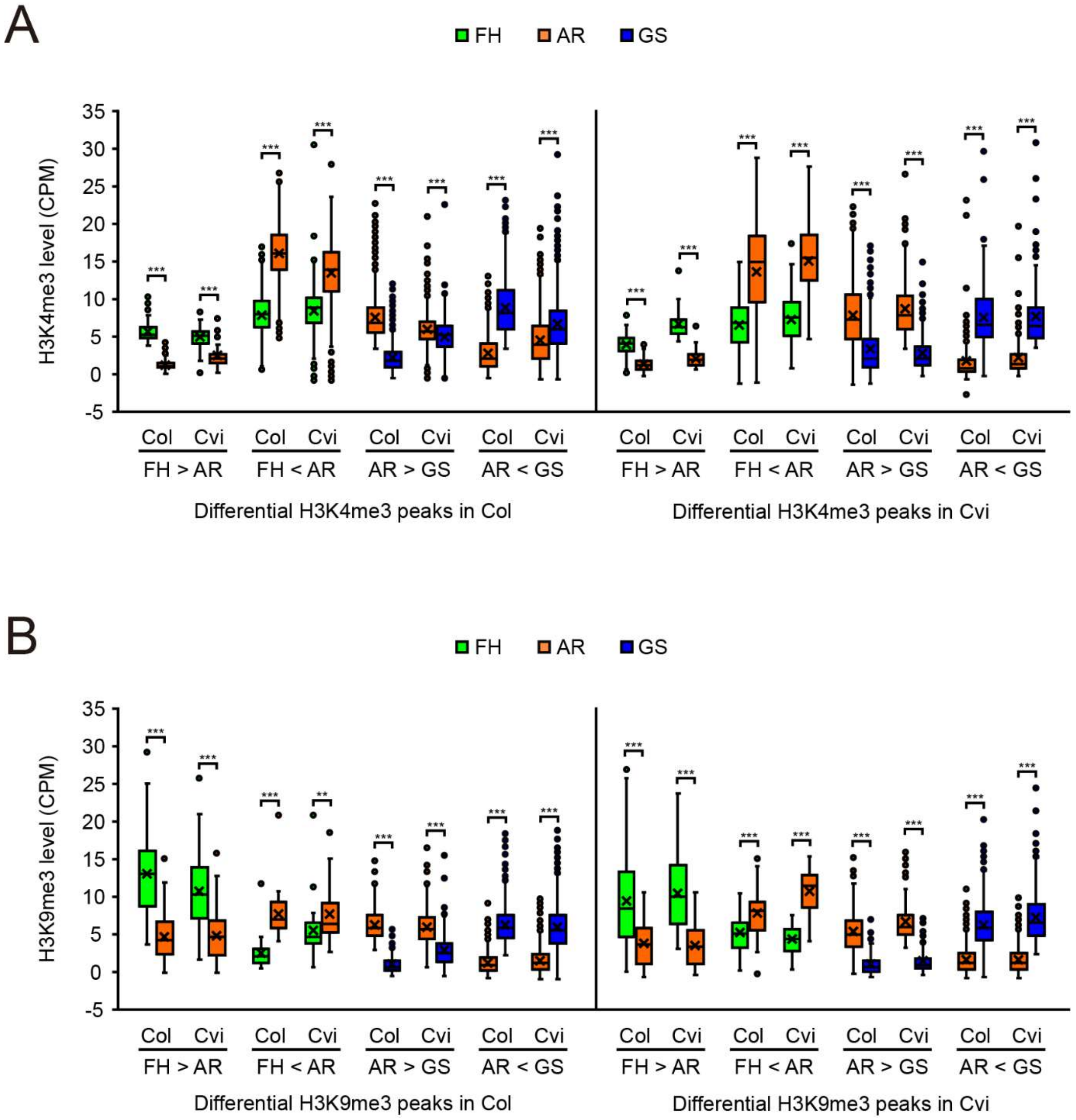

Supplementary Figure S6. Comparisons of H3K4me3 and H3K9me3 enrichment for differential peaks identified in Col and Cvi. (**A-B**) Boxplots showing levels of H3K4me3 (A) and H3K9me3 (B) in both Col and Cvi for H3K4me3 (A) and H3K9me3 (B) DMRs identified in Col (left) and Cvi (right). Statistical significance was evaluated using a two-tailed Mann–Whitney U test; $p < 0.001$ (***) and $p < 0.01$ (**).

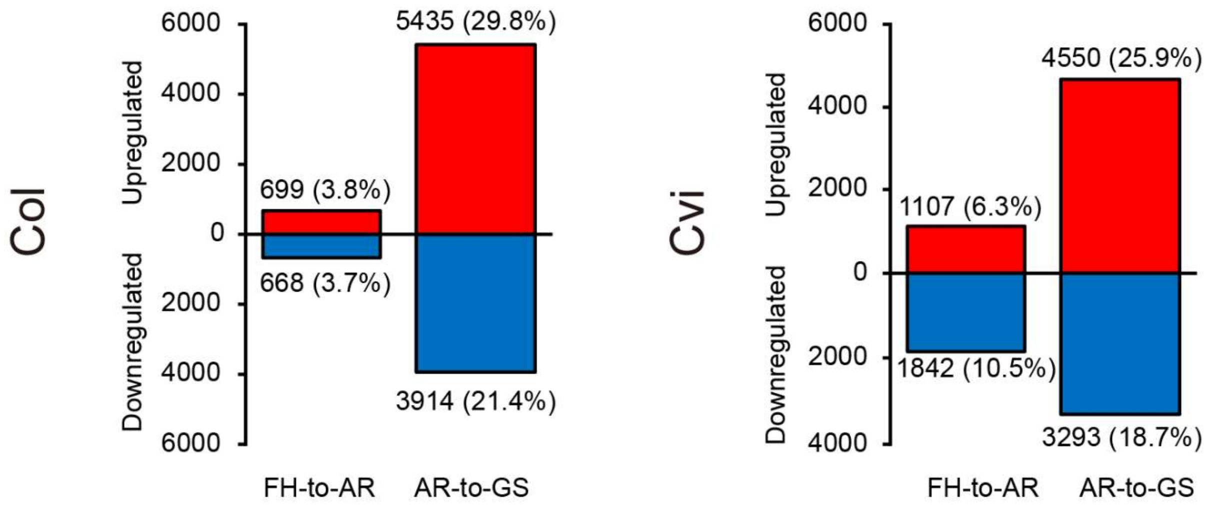

Supplementary Figure S7. DEGs during seed-state transitions. DEGs with increased or decreased expression during seed-state transitions are indicated in red or blue, respectively. The numbers of DEGs and their proportions relative to all expressed genes (indicated in parentheses) are shown above and below each bar.

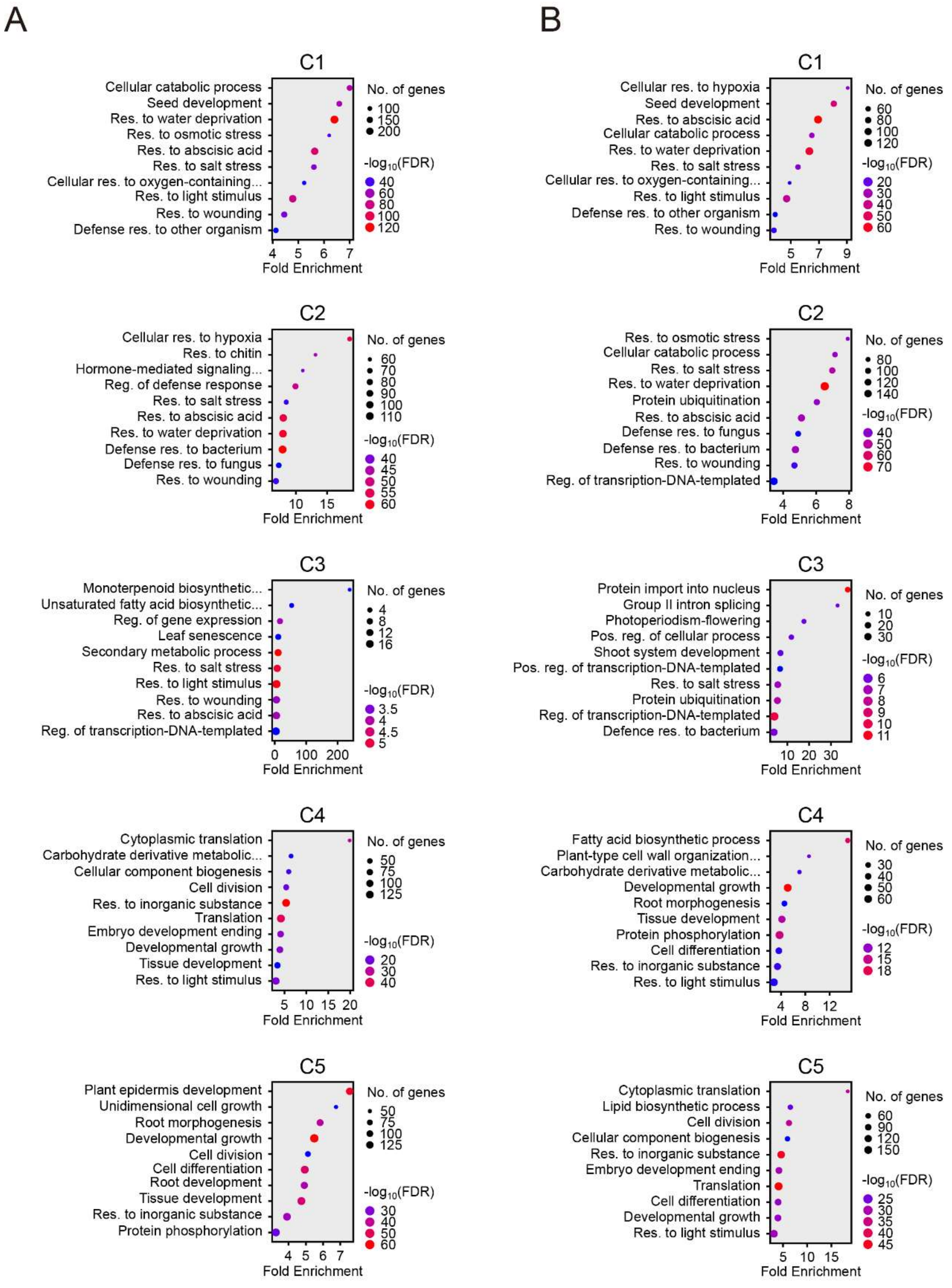

Supplementary Figure S8. GO enrichment analysis of clustered DEGs. (**A-B**) Dot plots showing enriched GO terms (biological processes; FDR < 0.05) for each DEG cluster (C1 to C5; see Figure 5) in Col (A) and Cvi (B). Dot size indicates the number of genes associated with each GO term, and dot color represents the $-\log_{10}$(FDR) value for enrichment.

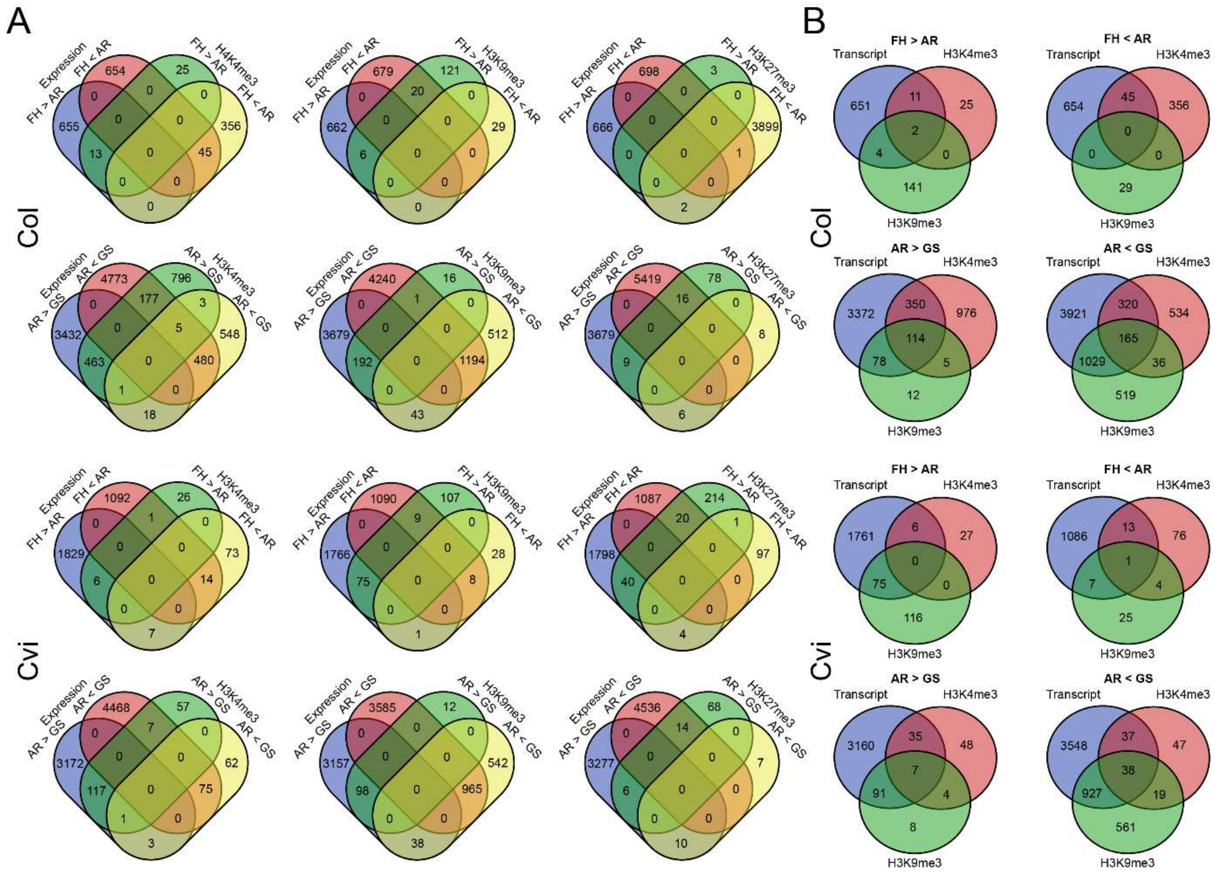

Supplementary Figure S9. Overlap analysis of differentially expressed genes (DEGs) and differentially methylated genes (DMGs). (**A**) Venn diagrams showing the overlaps between DEGs and DMGs during the FH-to-AR and the AR-to-GS transitions in Col (top two rows) and Cvi (bottom two rows). Left panels: H3K4me3; middle panels: H3K9me3; right panels: H3K27me3. (B) Venn diagrams showing the overlap among DEGs, DMGs for H3K4me3, and DMGs for H3K9me3 that show changes in the same direction —either all increased or all decreased— during each seed state transition.

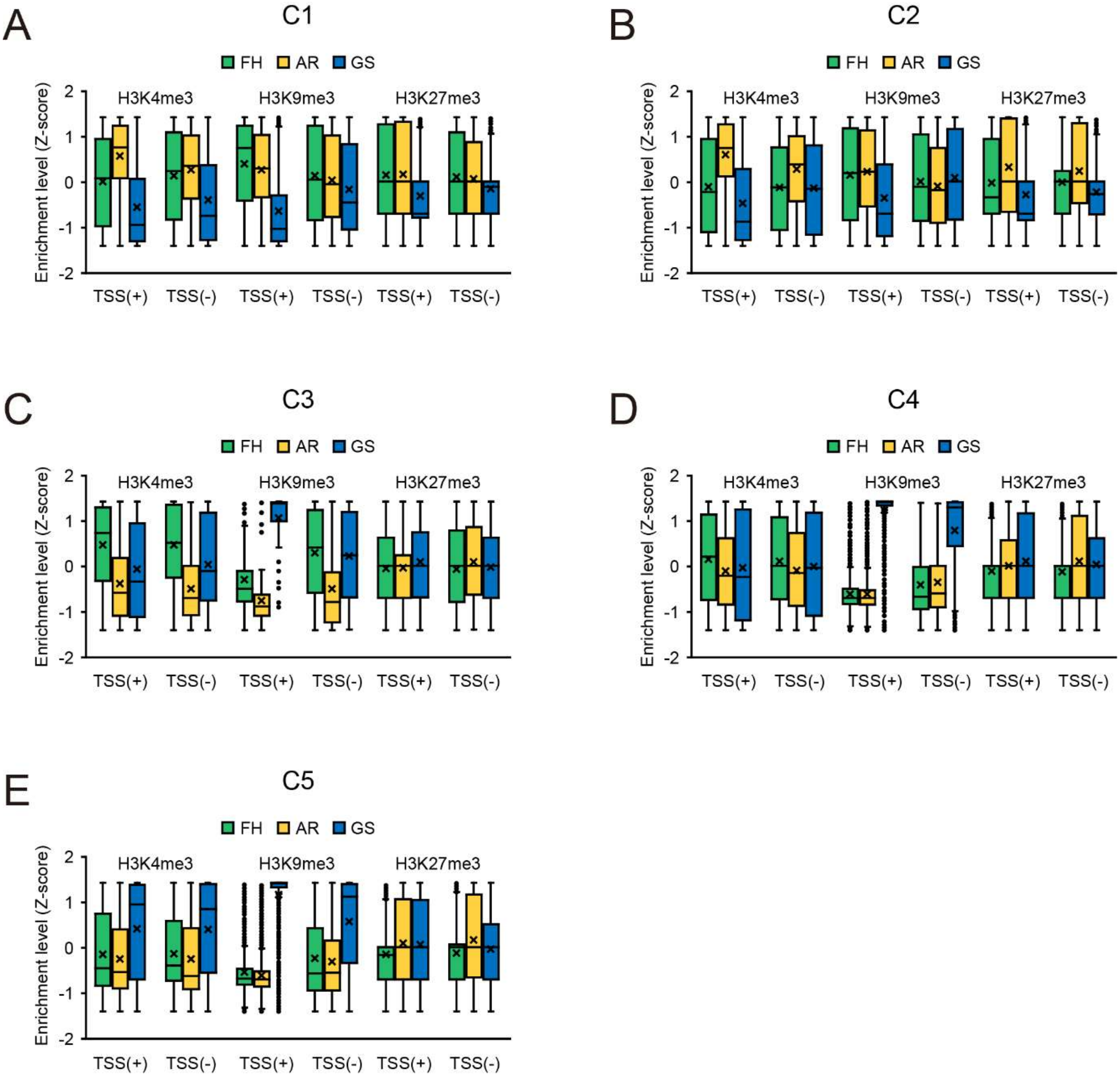

Supplementary Figure S10. Histone methylation dynamics in gene clusters with or without overlap with differentially expressed transcription start sites (DE TSSs) between AR and GS. (**A**–**E**) Z-scored enrichment levels of H3K4me3, H3K9me3, and H3K27me3 across FH, AR, and GS states for genes in clusters C1 to C5 as categorized by genes with DE TSS overlap (TSS(+)) or genes without DE TSS overlap (TSS(–)), which were identified by our analysis of published csRNA-seq data (see Materials and Methods). (A-B) Clusters C1 and C2 were analyzed for overlap with DE TSSs downregulated during the DS-to-L26 transition (comparable to AR > GS). (C-E) Clusters C3 to C5 were analyzed for overlap with DE TSSs upregulated during the DS-to-L26 transition (comparable to AR < GS).

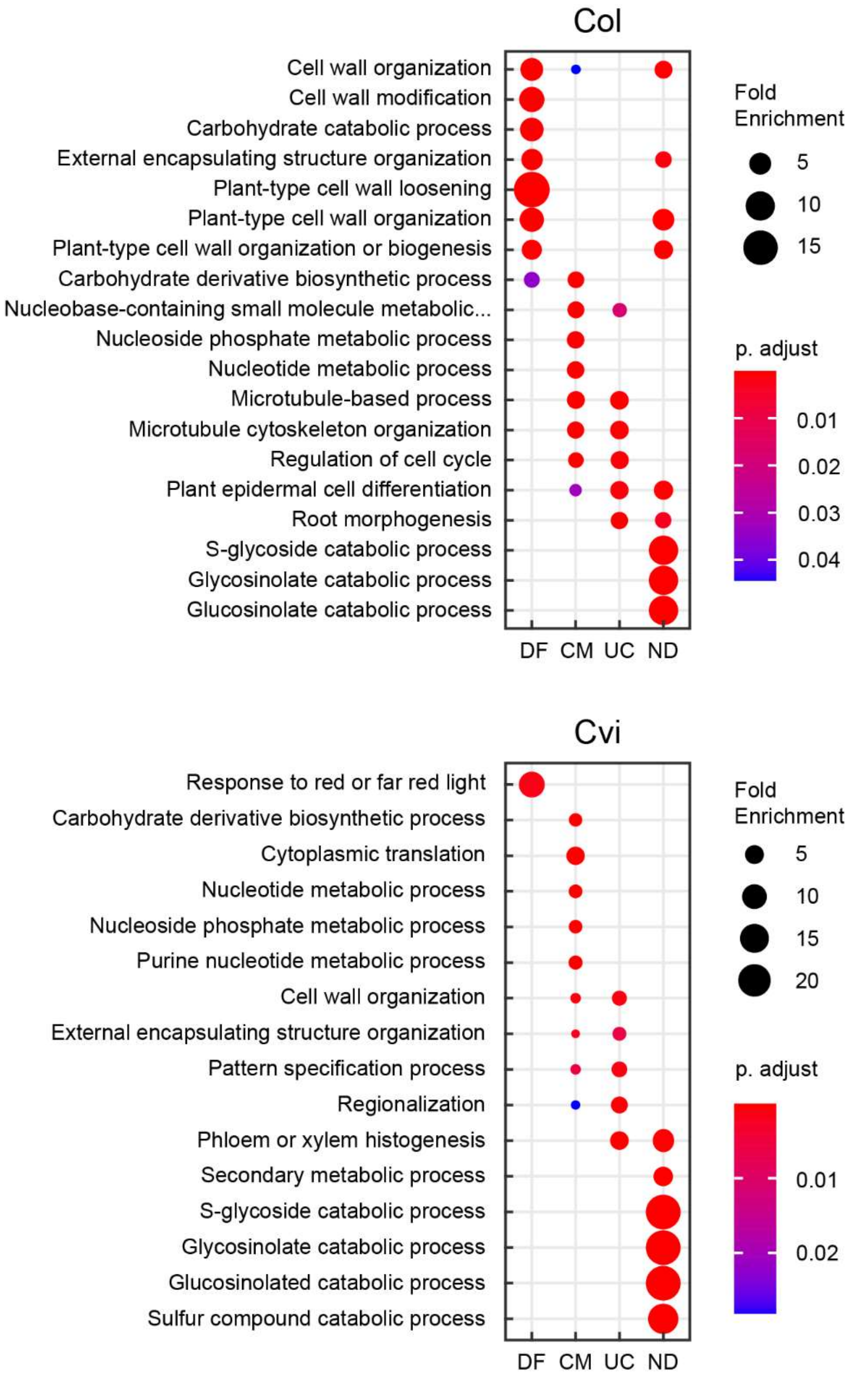

Supplementary Figure S11. GO analysis of genes in clusters C4 and C5 categorized by H3K4me3 status during the AR-to-GS transition. Dot plots displaying enriched GO terms (biological processes; FDR < 0.05) for each category defined in Figure 6. Dot size indicates the fold enrichment of genes associated with each GO term, and dot color reflects the FDR value for enrichment.

Supplementary tables

Supplementary Table S1. List of primers used in this study.

Primers for ChIP-qPCR

Name	Sequence (5' to 3')
AT1G05600 (EMB3101)_F	ATCTGATTCCTTGATGTGACCTG
AT1G05600 (EMB3101)_R	TACACCGAGCCATTGTGACC
AT1G12805_F	CAAATGGATGTCTTTATATCGGAAGAG
AT1G12805_R	ACCCGGAACTCATTATCTGGCG
AT1G13260 (RAV1)_F	AATGATGGTGTTTCTACGACGGG
AT1G13260 (RAV1)_R	CGTCCTCAAAGTTCAACAACACTCC
AT1G15550 (GA3OX1)_F	CGGATTCTTACAAGTGGACCC
AT1G15550 (GA3OX1)_R	GCACCCCAAGTTCTACATGC
AT1G27461 (DRG)_F	GTCAGACTCGTGAACTCCGCTTACAAG
AT1G27461 (DRG)_R	TCCTCCTTACCGTCTCTTCCCCA
AT1G68670 (HHO2)_F	ATAGATCTTGGTTCTTAGTCCC
AT1G68670 (HHO2)_R	GCTCTAAACATAAAGGAAGCTCG
AT1G75830 (PDF1.1)_F	CAGAAGCACCGATGGTGGTGG
AT1G75830 (PDF1.1)_R	GATCCATGTCGTGCTTTCTCAAGG
AT2G03520 (UPS4)_F	TTGGACAACAAGATAAACAAAGCCG
AT2G03520 (UPS4)_R	TTGTCTCAGCTTTATACTCGCTAGG
AT3G21295 (PWWP3)_F	CGTTTTCCTCTGTGAAGAAAGAGATTGAC
AT3G21295 (PWWP3)_R	ATCACTACTACCCATCACCCACC
AT3G24650 (ABI3)_F	CTAATCCCACCGTCCGAC
AT3G24650 (ABI3)_R	TCTGGCTGTGGCGGATAG
AT3G27870 (ALA8)_F	GCTGCTAATTTCATCCCAAAGTC
AT3G27870 (ALA8)_R	TCTTCTCCTCAAATCTTCAACACC
AT3G45310_F	CGAAGCTTTTGTTAGAGTAAAGAACAAG
AT3G45310_R	GTGAGTGAAGCGAGAAAAGGATAGAAC
AT4G17800 (AHL23)_F	TTCGACTGCGTTGCGACTTATGC
AT4G17800 (AHL23)_R	AACGTTCCTTGTAGCGTCACAACC
AT4G22505_F	ATGTCTCCTCCCATAACTCC
AT4G22505_R	GTGGTACTGAAGGTGTCCC
AT4G28520 (CRU3)_F	GCCAACCATGGGAAGGACAGGG
AT4G28520 (CRU3)_R	CACCTTCTGGTGCATGTCACGG
AT4G28530 (ANAC074)_F	ACTGTTGTTATTGTCGACGTAAAGC
AT4G28530 (ANAC074)_R	AAAATCCAAGTTTGTGATCAGTGG
AT5G11740 (AGP15)_F	GGCACAATCTGAGGCACCAGC
AT5G11740 (AGP15)_R	AGAGCTGAGCCAAAAACAAGAGCC
AT5G45830 (DOG1)_F	ATTACGCCGCAAAAAGAGCTG
AT5G45830 (DOG1)_R	TCGACAACCACCCATCCAAAT
AT5G65550_F	CTTCAGATAAGCTATGTGAGTCTCCG
AT5G65550_R	GGCCATACTGTGTCCTTCATCTCC
AT5G66400 (RAB18)_F	CGTCTTACCAGAACCGTCCAGG
AT5G66400 (RAB18)_R	TCCGTATCCTTGGCCACCTG

Primers for RT-qPCR

Name	Sequence (5' to 3')
AT1G69960 (PP2A)_RT_F	GTGATTATGTTGATCGAGGGT
AT1G69960 (PP2A)_RT_R	CCATACACTTGAGTAATTTGACGG
AT4G18650 (DOGL4)_RT_F	AGCGGTATCAGGTGGCTATG
AT4G18650 (DOGL4)_RT_R	ACTCAACGCATTGCGGTGGTG
AT4G31800 (WRKY18)_RT_F	CTACGTGCCTACTGAAACATCGG
AT4G31800 (WRKY18)_RT_R	TGCGTTGTACCTTCTTTTTTACTGG
AT5G45830 (DOG1)_RT_F	TTAAGTGCGGAGCAGCTAGC
AT5G45830 (DOG1)_RT_R	ACGGCGACGATCTCTCATAG
AT5G61610_RT_F	GGAGGAGTTGAGCATTTGTC
AT5G61610_RT_R	CCTGATTGCTTGCCACTTTC

Supplementary Table S2. Number of genes in clusters C4 and C5 categorized by H3K9me3 or H3K4me3 status during the AR-to-GS transition.

# of Genes (Ratio in %)	H3K9me3 status category				
	DF	CM	UC	ND	Total
Col C4 & C5	1167 (22.8 %)	623 (12.2 %)	2078 (40.6 %)	1245 (24.3 %)	5113 (100 %)
Cvi C4 & C5	937 (23.3 %)	614 (15.2 %)	1583 (39.3 %)	895 (22.2 %)	4029 (100 %)

# of Genes (Ratio in %)	H3K4me3 status catagory				
	DF	CM	UC	ND	Total
Col C4 & C5	465 (9.1 %)	2592 (50.7 %)	1110 (21.7 %)	946 (18.5 %)	5113 (100 %)
Cvi C4 & C5	70 (1.7 %)	2838 (70.4 %)	805 (20.0 %)	316 (7.8 %)	4029 (100 %)

Supplementary Table S3. Frequency tables of genes commonly regulated in Col and Cvi during the FH-to-AR transition. K4, H3K4me3; K9, H3K9me3.

# of Genes (Ratio in %)	Commonly downregulated genes ($Log_2(FC)$)			
	Col, K4	Col, K9	Cvi, K4	Cvi, K9
$Log_2(FC) \geq 1$	0	0	0	0
$0.5 \leq Log_2(FC) < 1$	1 (0.4 %)	0	2 (0.9 %)	0
$0.3 \leq Log_2(FC) < 0.5$	7 (3.1 %)	1 (0.4 %)	9 (4.0 %)	1 (0.4 %)

$-0.3 \le Log_2(FC) < 0.3$	120 (53.6 %)	176 (78.6 %)	138 (61.6 %)	118 (52.7 %)
$-0.5 \le Log_2(FC) < -0.3$	50 (22.3 %)	23 (10.3 %)	43 (19.2 %)	38 (17.0 %)
$-1 \le Log_2(FC) < -0.5$	39 (17.4 %)	16 (7.1 %)	30 (13.4 %)	53 (23.7 %)
$Log_2(FC) < -1$	7 (3.1 %)	8 (3.6 %)	2 (0.9 %)	14 (6.3 %)

# of Genes (Ratio in %)	Commonly upregulated genes ($Log_2(FC)$)			
	Col, K4	Col, K9	Cvi, K4	Cvi, K9
$Log_2(FC) \ge 1$	3 (3.0 %)	0	0	0
$0.5 \le Log_2(FC) < 1$	15 (15.2 %)	2 (2.0 %)	17 (17.2 %)	6 (6.1 %)
$0.3 \le Log_2(FC) < 0.5$	20 (20.2 %)	5 (5.1 %)	14 (14.1 %)	13 (13.1 %)
$-0.3 \le Log_2(FC) < 0.3$	59 (59.6 %)	80 (80.8 %)	63 (63.6 %)	70 (70.7 %)
$-0.5 \le Log_2(FC) < -0.3$	2 (2.0 %)	6 (6.1 %)	1 (1.0 %)	8 (8.1 %)
$-1 \le Log_2(FC) < -0.5$	0	6 (6.1 %)	4 (4.0 %)	2 (2.0 %)
$Log_2(FC) < -1$	0	0	0	0

# of Genes (Ratio in %)	Commonly non-differential genes ($Log_2(FC)$)			
	Col, K4	Col, K9	Cvi, K4	Cvi, K9
$Log_2(FC) \ge 1$	0	0	0	0
$0.5 \le Log_2(FC) < 1$	6 (2.7 %)	1 (0.4 %)	0	1 (0.4 %)
$0.3 \le Log_2(FC) < 0.5$	16 (7.1 %)	11 (4.9 %)	13 (5.8 %)	3 (1.3 %)
$-0.3 \le Log_2(FC) < 0.3$	180 (80.4 %)	203 (90.6 %)	188 (83.9 %)	197 (87.9 %)
$-0.5 \le Log_2(FC) < -0.3$	17 (7.6 %)	7 (3.1 %)	20 (8.9 %)	17 (7.6 %)
$-1 \le Log_2(FC) < -0.5$	5 (2.2 %)	2 (0.9 %)	3 (1.3 %)	6 (2.7 %)
$Log_2(FC) < -1$	0	0	0	0

Supplementary dataset list

Supplementary Dataset S1. Genomic coordinates and associated genes of H3K4me3, H3K9me3, and H3K27me3 peaks in Col.

Supplementary Dataset S2. Genomic coordinates and associated genes of H3K4me3, H3K9me3, and H3K27me3 peaks in Cvi.

Supplementary Dataset S3. Genomic coordinates and associated genes of differential histone methylation peaks during seed-state transitions in Col.

Supplementary Dataset S4. Genomic coordinates and associated genes of differential histone methylation peaks during seed-state transitions in Cvi.

Supplementary Dataset S5. Differential gene expression analysis of Col seed RNA-seq data.

Supplementary Dataset S6. Differential gene expression analysis of Cvi seed RNA-seq data.

Supplementary Dataset S7. GO enrichment analysis of clustered DEGs.

Supplementary Dataset S8. Row Z-scored histone methylation enrichment in clustered DEGs.

Supplementary Dataset S9. Differential expression analysis of seed csRNA-seq data.

Supplementary Dataset S10. GO enrichment analysis of genes in clusters C4 and C5 categorized by H3K9me3 or H3K4me3 status during the AR-to-GS transition.

Supplementary Dataset S11. Quantification of expression levels and enrichment of H3K4me3 and H3K9me3 in cluster C4 and C5 genes.

Supplementary Dataset S12. Quantification of expression levels and enrichment of H3K4me3 and H3K9me3 in DEGs shared between Col and Cvi or specific to Cvi during the FH-to-AR transition.

Supplementary Dataset S13. Functional annotation clustering of DEGs shared between Col and Cvi or specific to Cvi during the FH-to-AR transition.
